# Integrated analysis sheds light on evolutionary trajectories of young transcription start sites in the human genome

**DOI:** 10.1101/gr.231449.117

**Published:** 2018-05

**Authors:** Cai Li, Boris Lenhard, Nicholas M. Luscombe

**Affiliations:** 1 The Francis Crick Institute, London NW1 1AT, United Kingdom;; 2 Computational Regulatory Genomics, MRC London Institute of Medical Sciences, London W12 0NN, United Kingdom;; 3 Institute of Clinical Sciences, Faculty of Medicine, Imperial College London, London W12 0NN, United Kingdom;; 4 Sars International Centre for Marine Molecular Biology, University of Bergen, N-5008 Bergen, Norway;; 5 UCL Genetics Institute, University College London, London WC1E 6BT, United Kingdom;; 6 Okinawa Institute of Science & Technology Graduate University, Okinawa, 904-0495, Japan

## Abstract

Understanding the molecular mechanisms and evolution of the gene regulatory system remains a major challenge in biology. Transcription start sites (TSSs) are especially interesting because they are central to initiating gene expression. Previous studies revealed widespread transcription initiation and fast turnover of TSSs in mammalian genomes. Yet, how new TSSs originate and how they evolve over time remain poorly understood. To address these questions, we analyzed ∼200,000 human TSSs by integrating evolutionary (inter- and intra-species) and functional genomic data, particularly focusing on evolutionarily young TSSs that emerged in the primate lineage. TSSs were grouped according to their evolutionary age using sequence alignment information as a proxy. Comparisons of young and old TSSs revealed that (1) new TSSs emerge through a combination of intrinsic factors, like the sequence properties of transposable elements and tandem repeats, and extrinsic factors such as their proximity to existing regulatory modules; (2) new TSSs undergo rapid evolution that reduces the inherent instability of repeat sequences associated with a high propensity of TSS emergence; and (3) once established, the transcriptional competence of surviving TSSs is gradually enhanced, with evolutionary changes subject to temporal (fewer regulatory changes in younger TSSs) and spatial constraints (fewer regulatory changes in more isolated TSSs). These findings advance our understanding of how regulatory innovations arise in the genome throughout evolution and highlight the genomic robustness and evolvability in these processes.

Many studies have revealed that transcription is pervasive in prokaryotic and eukaryotic genomes (Clark et al. 2011; Wade and Grainger 2014). One recent study found that three-quarters of the human genome can be transcribed (Djebali et al. 2012), indicating a much more complex transcriptional landscape than previously thought. Transcription start sites (TSSs) are genomic loci where transcription initiates and so represent a critical class of regulatory elements for transcriptional control. Using high-throughput sequencing technologies, recent studies have greatly improved TSS annotations for many organisms, especially human, and uncovered new characteristics of transcriptional initiation (Core et al. 2008, 2014; The FANTOM Consortium 2014; Hon et al. 2017). An intriguing phenomenon of TSSs is their widespread occurrence throughout the genome, not only in stereotypical promoters of annotated genes but also in intergenic and intronic loci.

Many previous studies about TSS evolution focused on cross-species comparisons and revealed interesting macro-evolutionary patterns (Frith et al. 2006; Taylor et al. 2006; Yokoyama et al. 2011; Main et al. 2013; Young et al. 2015). By comparing human and mouse TSSs, a recent study found that >56% of protein-coding genes have experienced TSS turnover events since the species diverged (Young et al. 2015). Unlike macro-evolution, however, micro-evolutionary processes (i.e., intra-species evolution) of TSSs are relatively poorly understood. Given the high turnover rate of TSSs (Young et al. 2015), population genomic data could reveal a more detailed view of TSS evolution. Although some studies have made use of population genomic data, they pooled all TSSs to compare them with non-TSS elements (Ward and Kellis 2012) or focused on purifying selection (Scala et al. 2014; Young et al. 2015). Since different TSSs could have distinct evolutionary histories, pooling TSSs could bury interesting specific characteristics. A recent comprehensive study in *Drosophila melanogaster* populations investigated the relationship between genetic variations and TSS usage, identifying thousands of variants affecting transcript levels and promoter shapes, yielding important new insights into TSS evolution at the population level (Schor et al. 2017).

Despite extensive investigation, many questions about TSSs are yet to be addressed. Importantly, the evolutionary origin of new TSSs and evolutionary trajectories of newly emerged TSSs remain unresolved. Previous studies have suggested that repetitive sequences (repeats) are a rich source of new TSSs (Faulkner et al. 2009; Young et al. 2015), but the underlying mechanisms of how these sequences contribute to novel transcription initiation are underexplored. For instance, why do some repeats initiate transcription and not others? How does the host genome handle potential conflicts arising from the inherent instability of repeats associated with new TSSs? Furthermore, the subsequent changes of newly emerged TSSs and their evolutionary fates have not been systematically investigated. Only by addressing these questions can we begin to understand how regulatory innovations arise in the genome and how they contribute to biological diversity and adaptation.

Here, to gain detailed insights into young TSSs in the human genome, we integrated cross-species evolutionary and functional genomic data to investigate the origin of new TSSs and their subsequent evolution.

## Results

### Identifying evolutionarily young TSSs in the human genome

Using the Cap Analysis of Gene Expression (CAGE) sequencing technology, the FANTOM5 project (The FANTOM Consortium 2014) generated the most comprehensive TSS annotation for the human genome to date, covering major primary cell and tissue types. To identify evolutionarily young TSSs, we started with the “robust” data set from FANTOM5 comprising 201,873 high-confidence TSSs. After filtering TSSs that could confound downstream analysis (see Methods), we grouped the remaining 151,902 TSSs according to their inferred evolutionary ages. Since we lack large-scale CAGE data sets for other primate genomes, it is impossible to perform direct cross-species comparisons of TSS annotations. However, previous studies revealed that sequence-intrinsic properties of many promoters can drive transcription initiation autonomously (Nguyen et al. 2016; van Arensbergen et al. 2017), indicating that sequence is an important determinant of their functional capacity. Moreover, 80% of human TSSs that can be aligned to an orthologous sequence were found to have detectable transcriptional initiation in the mouse genome (Young et al. 2015). Therefore, we used the sequence homology as a proxy for the evolutionary age of TSSs.

**Figure 1.**
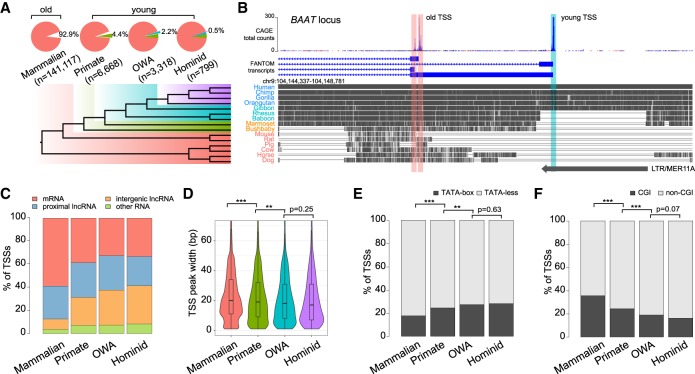
Classification of human transcription start sites (TSSs) by evolutionary age. (*A*) (*Top*) Statistics of four TSS groups defined by sequence age using genomic alignments. (*Bottom*) Phylogeny with colors indicating the corresponding evolutionary age of each group. OWA = Old World anthropoids. (*B*) Example *BAAT* locus containing two “mammalian” TSSs (“old”; red shade) and one “OWA” TSS (“young”; cyan shade). (*Top*) FANTOM5 CAGE data and annotations indicating different TSSs. (*Middle*) Multiple genome alignments with gray blocks representing regions of sequence homology in different species. (*Bottom*) An annotated long tandem repeat (LTR) element overlapping with the young TSS. (*C*) Composition of associated transcript types in each TSS group. (*D*) Violin-box plots for TSS peak widths of each TSS group. (*E*) Proportions of TATA-box-containing and TATA-less TSSs. (*F*) Proportions of CGI-associated and non-CGI-associated TSSs. Statistical significances in *D* were calculated by one-tailed Wilcoxon rank-sum tests; statistical significances in *E* and *F* by Fisher's exact tests; (**) *P* < 0.01, (***) *P* < 0.001.

We examined sequence alignments of human TSSs with 16 other genomes (10 representing major primate lineages and six nonprimate mammals as outgroups). Based on the presence or absence of alignable sequences, we categorized human TSSs into four groups (Fig. 1A; see Methods and Supplemental Tables S1–S3 for more details): 141,117 TSSs in the “mammalian” group (92.9% of all TSSs); 6668 (4.4%) in the “primate” group; 3318 (2.2%) in the “OWA” (Old World anthropoids) group; and 799 (0.5%) in the “hominid” group. To check the suitability of this approach, we assessed available CAGE-defined TSSs in four other mammals (macaque, mouse, rat, and dog) from FANTOM (Francescatto et al. 2017; Lizio et al. 2017). Applying the same criteria, we found that a large majority of nonhuman TSSs that can be aligned to the human genome have a detectable transcription initiation signal within the corresponding locus (Supplemental Fig. S1), indicating that sequence homology is a reasonable proxy for TSS age.

The relatively large numbers of TSSs in the different evolutionary groups enable us to perform detailed comparative analysis. We consider those in the “mammalian” group to be evolutionarily old TSSs and those in the three other groups to be young (Fig. 1A). For example, in the *BAAT* locus shown in Figure 1B, there are two old TSSs present in both primate and nonprimate mammalian genomes and one young TSS established during the evolution of OWAs. The young TSS is located in a region overlapping a long terminal repeat (LTR) element (Fig. 1B), suggesting that it originated from an LTR insertion event.

We first examined some general features among TSS groups. Old TSSs are mainly associated with mRNAs (59%), whereas many young TSSs are associated with long noncoding RNAs (lncRNAs, 54%–60%) (Fig. 1C). The proportion of mRNA-associated TSSs increases with age and vice versa for the intergenic lncRNA TSSs (Fig. 1C). Relative to older TSSs, younger ones generally have narrower CAGE TSS peaks (Fig. 1D) and comprise more TATA-box-containing promoters (Fig. 1E) and fewer CpG island (CGI)-associated promoters (Fig. 1F). This is consistent with previous observations about broad and sharp TSSs in mammalian genomes (Lenhard et al. 2012; The FANTOM Consortium 2014), which found that CGI promoters are usually associated with broad TSSs and housekeeping genes, whereas TATA-box promoters contain sharp TSSs and are associated with tissue-specific genes. Both old and young TSSs exhibit elevated GC and CpG content compared with flanking regions (Supplemental Fig. S2), and old TSSs tend to be more GC-rich relative to young ones. We also found that the “hominid” TSS group has higher average GC and CpG content relative to “OWA” and “primate” groups (Supplemental Fig. S2), which could be partly due to fewer historical deamination events of methylated cytosines in very young TSS loci (see below).

### Intrinsic factors within repetitive sequences contribute to novel TSSs

Next, we systematically investigated how new TSSs originate and evolve over time. Earlier FANTOM projects showed that many mammalian transcripts initiate within repetitive elements, especially retrotransposons (Faulkner et al. 2009; Fort et al. 2014; Young et al. 2015). Given the extensive transposition that occurred during mammalian evolution, transposable elements (TEs, including retrotransposons and DNA transposons) could be an important source of novel TSSs. In addition, tandem repeats are abundant in promoter regions and have significant impact on gene expression (Sawaya et al. 2013; Bilgin Sonay et al. 2015).

**Figure 2.**
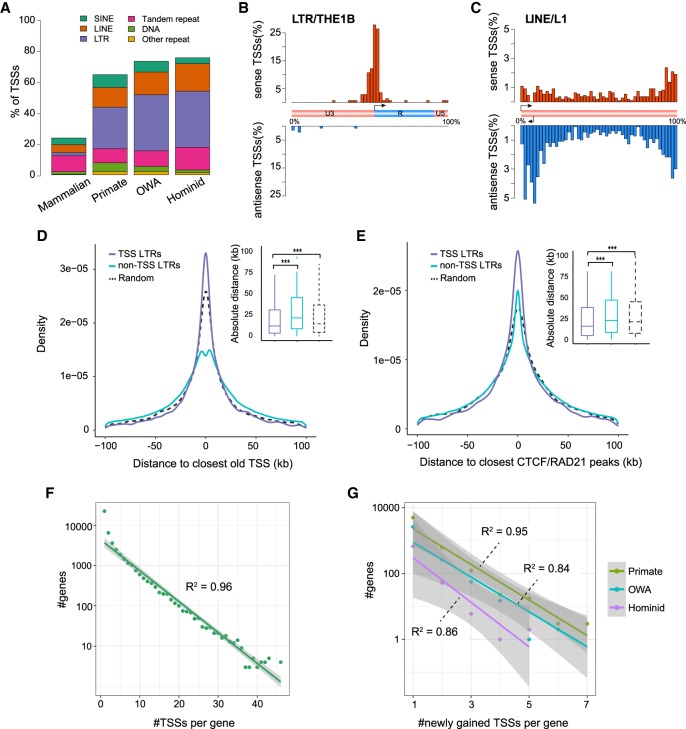
Intrinsic and extrinsic factors contributing to the origin of new TSSs. (*A*) Composition of major repeat families in four TSS groups. We considered the nearest repeat element within TSS ± 100 bp. (*B*) Distribution of young TSSs plotted against the consensus LTR/THE1B element. Schematic of THE1B indicates the original TSS, U3, R, and U5 regions for the element. (*C*) Distribution of young TSSs plotted against the consensus LINE/L1 element. Schematic of the L1 structure indicates the original sense and antisense TSSs at the 5′ end. (*D*) Comparison of distances of TSS-associated and non-TSS-associated LTRs to the closest old TSSs. Distances of random intervals to the closest old TSSs are also provided for comparison. *Inset* shows a box plot of the same distribution. (*E*) Comparison of distances of TSS-associated and non-TSS-associated LTRs to the closest CTCF or RAD21 ChIA-PET peaks (from GM12878; only mammalian-conserved peaks were used). Distances of random intervals are calculated in a similar manner to panel *D*. *Inset* shows a box plot of the same distribution. (*F*) Exponential approximation for the number of genes with a certain number of TSSs and number of TSSs per gene, based on data of all TSSs. *R*^2^ is the coefficient of determination for the linear regression. Gray shade indicates the 95% confidence interval. (*G*) Exponential approximation for number of genes and number of newly gained TSSs per gene, based on data of newly emerged TSSs in three periods. Statistical significances in *D* and *E* were calculated by one-tailed Wilcoxon rank-sum tests; (***) *P* < 0.001.

We examined the relationship between TSSs and repeats based on annotations from RepeatMasker (Tarailo-Graovac and Chen 2009), TRF (Benson 1999), and STRcat (Willems et al. 2014). We found that ∼70% of young TSSs have at least one repeat element within ±100 bp, but that only 24% of old TSSs do (Fig. 2A). Whereas 43% of repetitive sequences associated with old TSSs are tandem repeats, 52% of young TSSs are associated with retrotransposons, including LTRs, long interspersed nuclear elements (LINEs), and short interspersed nuclear elements (SINEs) (Fig. 2A). Because some tandem repeats are derived from retrotransposons, we performed an alternative analysis considering only the nearest retrotransposons of TSSs (Supplemental Table S4; Supplemental Fig. S3). LTRs are the most abundant retrotransposon class, associated with ∼30% of young TSSs. Fourteen percent and 8% of young TSSs are associated with LINEs and SINEs, respectively. Compared with expectation, young TSSs (“OWA” and “hominid,” but not “primate”) are enriched in LTRs but depleted in LINEs and SINEs (Supplemental Fig. S4), implying that LTR insertions are more likely to create new TSSs. The large number of retrotransposons associated with young TSSs suggests a major role of retrotransposition in forming new TSSs.

Faulkner et al. (2009) revealed that, surprisingly, many TE-derived TSSs do not appear in the canonical 5′ promoters of TEs but are unevenly distributed along TE consensus sequences (Faulkner et al. 2009); however, how these TE-derived sequences contribute to transcription initiation was not discussed in detail. To gain more insight, we mapped TSSs to TE consensus sequences: The distributions we obtained are similar to those found by Faulkner et al. (2009) but also reveal some new differences which are likely due to upgraded CAGE protocols (Kanamori-Katayama et al. 2011), improvements in the TSS-calling method (Hon et al. 2017), and other subtle differences in our analyses.

TSSs associated with LTRs are mainly in the sense strand and cluster within narrow regions (see Fig. 2B for the THE1B subfamily and Supplemental Fig. S5 for more subfamilies). Since the consensus LTR sequences contain promoters for endogenous retroviral elements (ERVs), the sense-biased distributions of TSSs suggest that initiation events in these regions are mainly contributed by the original ERV promoter activity within LTRs. These patterns were not observed in Faulkner et al. (2009), as they investigated the distributions of TSSs along LTR superfamilies but not the subfamilies. Our subfamily-level analysis offered insight into sequence determinants of these LTR-derived TSSs. We also found that ∼50% of young LTR-associated TSSs contain a TATA-box motif 25–35 bp upstream (Supplemental Fig. S6)—a TATA-box motif upstream of the TSS is found in many but not all LTR consensus sequences—whereas the proportion drops to ∼30% for old LTR-associated TSSs. This suggests that a substantial fraction of TATA-less promoters may have originated as LTR-derived TATA-box-containing promoters.

LINE-1 (L1) is the most abundant LINE family, covering ∼20% of the human genome. The overall distribution of TSSs along L1 elements (Fig. 2C) is similar to that of Faulkner et al. (2009); however, there are further observations for different L1 subfamilies (Supplemental Fig. S7). For some (e.g., L1PB1, L1PBa1), transcription initiation occurs mainly at the 5′ end of antisense promoters (which were discussed by Faulkner et al. 2009), whereas for others, initiation occurs at the 3′ end (e.g., L1MB7) or rather sporadically (e.g., L1M4). Although the background frequencies of remnant sequences of L1 subfamilies in the human genome can explain such differences to some degree, it is apparently not the only reason (Supplemental Fig. S7). This suggests that different sequences from different L1 subfamilies have a very variable propensity to drive transcription initiation, which might be associated with the frequent recruitment of novel regulatory regions during L1 evolution (Khan et al. 2006).

*Alu* elements comprise the most abundant SINE family, covering ∼10% of the human genome. Although *Alu*s are frequently inserted in promoter-proximal and intronic regions, previous research found that they generally lack the capacity to drive autonomous transcription (van Arensbergen et al. 2017). In the FANTOM5 data set, we observed many new TSSs located around the 3′ poly(A) region and the A-rich linker region of *Alu*s, which we believe probably arose from technical artifacts in the CAGE method (Supplemental Fig. S8; see Methods for more details). The remaining TSSs tend to be enriched at the 5′end of *Alu*s in the antisense strand (Supplemental Fig. S8), but how these sequences help drive transcription initiation is unclear.

Nine percent of young TSSs contain tandem repeats that are not associated with TEs. Unlike those derived from new TE insertions, the flanking regions of these tandem repeats tend to be conserved among mammals and have higher GC content (Supplemental Fig. S9). This suggests that some new TSSs in these regions are likely due to autonomous expansions of tandem repeats located proximally to pre-existing promoters (some examples are provided in Supplemental Fig. S9). Consistent with previously reported enrichment of tandem repeats in primate promoters (Sawaya et al. 2013; Ohadi et al. 2015; Young et al. 2015; Gymrek et al. 2016), the expansions of these repeats might have duplicated or changed pre-existing regulatory signals, which helped create new TSSs.

### Extrinsic factors contribute to novel TSSs

The vast majority of repeats harboring proto-TSS sequences do not exhibit initiation signals; for instance, <1% of human LTRs are associated with CAGE-defined TSSs. This suggests that additional extrinsic factors dictate whether transcription initiation can occur in these regions. A well-known major factor is that transcription from repeats tends to be highly suppressed by the host via mechanisms such as DNA methylation and H3 lysine 9 methylation (Slotkin and Martienssen 2007). In addition, we reasoned that the proximity of proto-TSSs to established transcriptional units might promote novel initiation by allowing access to the existing transcriptional machinery. To test this hypothesis, we examined the genomic distances of LTRs to old TSSs: We found that LTRs containing young TSSs tend to be closer to established TSSs compared with LTRs lacking TSSs (Fig. 2D). We also examined published ChIA-PET data sets that identified spatially proximal regulatory regions in the nucleus. We focused on CTCF and RAD21 data sets (Grubert et al. 2015; Tang et al. 2015), which are important for chromatin architecture and linking regulatory modules for transcriptional regulation. CTCF-binding sites were also found to be highly conserved during evolution (Merkenschlager and Odom 2013). Examining the distances of LTRs to mammalian-conserved ChIA-PET interaction loci (see Methods), we found that TSS-associated LTRs are closer to CTCF- or RAD21-bound loci compared with nonassociated LTRs (Fig. 2E). We suggest that the proximity to such loci might enable those proto-TSSs to enlist assembled transcriptional machinery of other transcriptional units.

The spatial proximity of young and old TSSs may also help to explain the exponential decrease in the number of genes with a certain number of TSSs (Fig. 2F). The distribution indicates that most genes have few TSSs, whereas a small fraction of genes have large numbers of TSSs. A similar relationship is also seen for newly emerged TSSs (Fig. 2G), which implies that a small number of genes gain many new TSSs during a specific period. The exponential relationship is independent of gene lengths (Supplemental Fig. S10). We also observed a positive correlation between the number of pre-existing TSSs per gene and the number of newly gained TSSs per gene (Pearson's *r* = 0.24, *P* < 2.2 × 10^−16^) (Supplemental Fig. S11). Based on these observations, we suggest that most new repeat-derived TSSs arise opportunistically because of sequence-intrinsic properties as well as their proximity to existing transcriptional units. As time goes by, a proportion of these new TSSs are exapted by proximal genes to form alternative promoters. These observations also suggest that the existing transcriptional landscape to some extent constrains the emergence and evolution of new TSSs.

### Young TSSs undergo rapid sequence evolution

**Figure 3.**
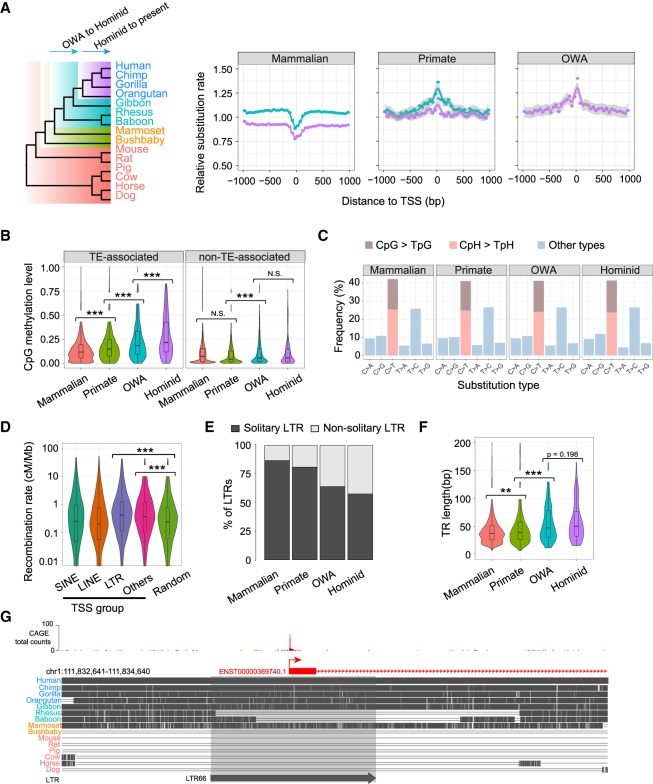
Rapid sequence evolution of young TSSs. (*A*) (*Left*) Phylogeny of genomes used for evolutionary rate analysis, with arrows indicating the two considered periods. (*Right*) Distributions of relative substitution rates (normalized by genomic average) inferred from genomic alignments for three TSS groups using 50-bp bins along TSS ± 1 kb. The curve colors correspond to the two periods highlighted in the phylogeny. Best-fit curves were estimated by “loess,” and gray shades indicate 95% confidence intervals. (*B*) Violin-box plots for germline DNA methylation levels (a male germline data set from Guo et al. 2015) for different TSS groups. For each TSS, the average methylation level of CpGs was calculated for TSS ± 1 kb. (*C*) Frequencies of nucleotide substitution types in different TSS groups, based on the data from the 1000 Genomes Project. (*D*) Violin-box plots of recombination rates among TSSs associated with different types of retrotransposons and random genomic background. The recombination rate of each TSS was defined as the average rate for TSS ± 1 kb. Background recombination rates were generated for randomly selected 2-kb windows in the human genome. (*E*) The fraction of solitary LTRs in four TSS groups. (*F*) Violin-box plots of tandem repeat (TR) lengths in the four TSS groups. (*G*) Genome browser view depicting a putative TSS death event around an LTR66 element in the lineages of rhesus and baboon. Statistical significances in *B*, *D*, and *F* were calculated by one-tailed Wilcoxon rank-sum tests. (**) *P* < 0.01, (***) *P* < 0.001, (N.S.) not significant.

Having established the emergence of new TSSs, next we investigated the subsequent sequence changes that TSSs undergo once they have appeared in the genome. We performed this by examining inferred sequence changes for two recent evolutionary periods compared with the genomic background: from the last common ancestor (LCA) of OWAs to the LCA of hominids, and from the LCA of hominids to the present (Fig. 3A; see Methods for more details). We calculated the relative rates of substitutions and small insertions/deletions, normalized by the genomic average. Positions proximal to old TSSs have lower substitution rates compared with the surrounding regions and the genomic average (Fig. 3A), suggesting they were subject to purifying selection in both evolutionary periods. In contrast, proximal positions of young TSSs display elevated substitution rates (Fig. 3A), suggesting that young TSS loci experienced rapid sequence evolution. For “primate” TSSs, the substitution rates during the OWA-to-hominid period are higher than in the hominid-to-present period (Fig. 3A), suggesting that newly emerged TSSs evolve rapidly at first and then at a slower rate later. These patterns are not observed in the insertion/deletion rates (Supplemental Fig. S12); we speculate that this might be due to saturated insertion/deletion mutations (i.e., excessive independent mutations in multiple species at a same site) and ancestral insertion/deletion events not being accurately inferred using alignments of extant species. Additionally, by examining the population polymorphism data from the 1000 Genomes Project, we found that young TSSs also have elevated variant densities relative to surrounding regions (Supplemental Fig. S13), further supporting that young TSSs undergo rapid sequence evolution.

### Endogenous mutational processes contribute to rapid evolution of young TSSs

We then asked in what way young TSSs evolve after appearing in the genome. Since many young TSSs are associated with repetitive sequences, we reasoned that mutational processes associated with repeats could contribute to the observed rapid evolution.

One contributing factor could be DNA methylation, which is one of main mechanisms for repressing TE activities (Slotkin and Martienssen 2007). Younger TSSs have significantly higher levels of CpG methylation in the germline compared with older TSSs (Fig. 3B; Supplemental Fig. S14). In addition, TE-associated TSSs tend to have higher levels of CpG methylation compared with non-TE TSSs (Fig. 3B). Because methylated cytosine (mC) can frequently be deaminated to thymine (T), DNA hypermethylation around young TSSs in the germline represents an important contributor for the elevated substitution rates. This is further supported by the substitution patterns in the human polymorphism data, in which the C > T is the most common substitution type (∼40% of all substitutions) in all TSS groups and ∼17% of mutations occur in the CpG context (Fig. 3C).

Another contributing factor is recombination, which is associated with mutations and GC-biased gene conversion (Pratto et al. 2014). We found that LTR-associated TSSs have significantly higher recombination rates relative to the genomic average (Fig. 3D). Higher recombination rates are also observed in non-TE-associated young TSSs (Fig. 3D). Consistently, older LTR-associated TSSs have more solitary LTRs (“solitary” means that the internal parts of ERVs have been deleted) (Fig. 3E), which are known to result from allelic or nonallelic homologous recombination (Chuong et al. 2017). As recombination hotspots evolve rapidly (Baudat et al. 2013) and ancient recombination events are difficult to detect, it is possible that recombination also contributed to the rapid evolution of SINE/LINE-associated TSSs.

A third contributing factor is the instability of tandem repeats. Previous research showed that the mutability of microsatellites (also known as short tandem repeats) increases with their lengths and long microsatellites tend to be shortened or interrupted by mutations over time (Kelkar et al. 2008; Eckert and Hile 2009). Indeed, we found that tandem repeats associated with younger TSSs tend to be longer than those in older TSSs (Fig. 3F), implying that they are more likely to mutate.

### Consequences of rapid evolution in young TSSs

A direct consequence of the rapid evolution around young TSSs is a more stable genomic environment, since mutations could reduce or eliminate the transposition capacity of TEs or the mutability of tandem repeats around TSSs. Therefore, these mutational processes probably help resolve genomic conflicts caused by inherent instability of associated repeats around young TSSs. We suspect some sequence changes may lead to the death of some young TSSs by disrupting critical promoter components required for transcription initiation. An example is shown in Figure 3G, in which an LTR locus with transcription initiation signals in human has been deleted from rhesus and baboon. Large-scale CAGE-defined TSS data sets in other primate species will enable the analysis of the evolutionary death of young TSSs.

### TSSs of different evolutionary ages exhibit distinct functional signatures

Previous comparison between human and mouse CAGE-defined TSSs revealed that lineage-specific TSSs tend to have tissue-restricted expression profiles, often in samples associated with testis, immunity, or brain (Young et al. 2015). However, the evolutionary trajectories in which the regulatory functions of these lineage-specific TSSs are established remain unclear.

**Figure 4.**
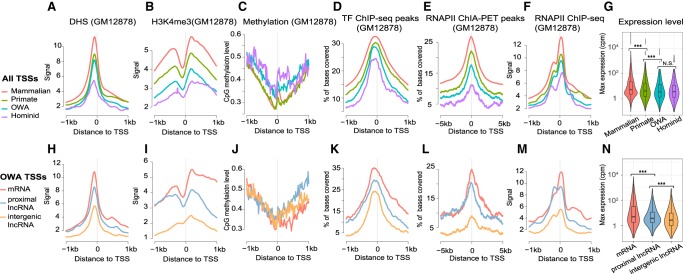
Distinct functional signatures in different TSS groups. (*A*) Metaprofiles of DHS signals for four TSS groups using 20-bp bins along TSS ± 1 kb (same bin sizes for other panels). (*B*) Metaprofiles of H3K4me3 signals. (*C*) Metaprofiles of CpG methylation levels. (*D*) Metaprofiles of coverage ratio by TF ChIP-seq peaks. Previously called peaks of 88 TF ChIP-seq data sets from ENCODE were merged, and for every bin of each TSS locus we calculated the proportion of bases covered by merged peaks. (*E*) Metaprofiles of coverage ratio by RNAP II ChIA-PET peaks. (*F*) Metaprofiles of RNAP II ChIP-seq signals. (*G*) Violin-box plots of maximum expression levels of TSSs across primary cell samples, based on the data from FANTOM. (*H*–*N*) As in *A*–*G*, but specifically for the “OWA” TSS subgroups of different transcript types. All functional genomic data except the expression data are for the GM12878 cell line.

We compared functional genomic profiles between TSS groups, including DNase I hypersensitivity (DHS), histone modifications, DNA methylation, transcription factor (TF) binding, and chromatin interactions. TSSs of different ages exhibit segregating functional signatures (see Fig. 4 for GM12878 cell line) that are consistently maintained across different cell lines (see Supplemental Fig. S15 for K562 and H1-hESC cell lines). Compared with older TSSs, younger TSSs tend to have lower chromatin accessibility (DHS) (Fig. 4A), lower levels of activating histone modifications (e.g., H3K4me3, H3K27ac, H3K4me1, and H3K9ac) (Fig. 4B; Supplemental Fig. S16) and higher CpG methylation (Fig. 4C), suggesting younger TSSs exist in more repressed chromatin environments. By examining ChIP-seq data for TFs from ENCODE, we found that older TSS loci tend to display more binding (i.e., more surrounding sequences overlapping ChIP-seq peaks) relative to younger TSSs (see Fig. 4D and Supplemental Fig. S17 for metaprofiles of individual TFs). We also observed a similar trend for computationally predicted TFBSs (Supplemental Fig. S18). We further analyzed ChIA-PET interaction data for RNA polymerase II (RNAP II), usually formed within CTCF/cohesin looped structures and considered to reflect promoter-enhancer interactions (Tang et al. 2015). We found that younger TSSs have fewer RNAP II chromatin interactions compared to older TSSs (Fig. 4E), suggesting that younger TSSs tend to lack connections to other regulatory modules. As for transcriptional output, younger TSSs tend to display lower expression than older TSSs (Fig. 4F,G), which is consistent with a previous observation from the human-mouse comparison that lineage-specific promoters tend to have lower expression levels (Young et al. 2015). Taken together, these observations indicate that the evolution of TSSs leaves footprints in the functional signatures: namely, that younger TSSs tend to have smaller regulatory impact on a genome and that the impact increases with time.

We also observed heterogeneity of functional signatures within TSS groups. Within an age-group, TSSs associated with mRNAs tend to have higher DHS, more activating histone modifications, more TF-binding, and more spatial interactions than other TSSs (Fig. 4H–M; Supplemental Fig. S19), indicating that they are more transcriptionally active. Consistently, mRNA TSSs tend to have higher expression levels than other TSSs within the same group (Fig. 4N). Furthermore, TSSs of lncRNAs that are proximal to annotated genes are more transcriptionally active compared with those of intergenic lncRNAs, likely because they are close to other transcriptional units. Overall, these findings suggest that the locations of young TSSs relative to existing functional genomic regions influence their regulatory impact.

### Evolution of regulatory functions of young TSSs appears to be subject to temporal and spatial constraints

The segregating functional signatures of different TSS categories strongly imply that the regulatory outcomes of young TSSs gradually change over time. The regulatory impact of historical and fixed sequence changes around TSSs is difficult to assess; however, there are many ongoing changes around TSSs within human populations whose regulatory effects have been widely studied by combining functional and population genomics (Albert and Kruglyak 2015). Two common strategies are to identify regulatory quantitative trait loci (rQTLs; e.g., TF-binding QTLs, histone modification QTLs) and variants associated with regulatory allelic specificities (AS; e.g., allele-specific TF binding, allele-specific methylation). Although no QTL or AS study has been specifically performed for human CAGE-defined TSSs, we can apply data from genome-wide rQTL and AS studies of relevant molecular traits. A previous study (Cheng et al. 2012) revealed that expression levels of CAGE-defined TSSs are highly correlated with other functional signatures such as TF-binding, histone modifications, and DHS in surrounding regions and can be largely predicted by those functional signatures (*R*^2^ > 0.7). Therefore, we reasoned that changes in the regulatory outcomes of TSSs can be approximated by changes in related functional signatures in surrounding regions. By examining rQTLs and AS variants (together called regulatory variants) in TSS loci of different ages, we can gain insights into the tempo and mode of regulatory evolution of TSSs.

**Figure 5.**
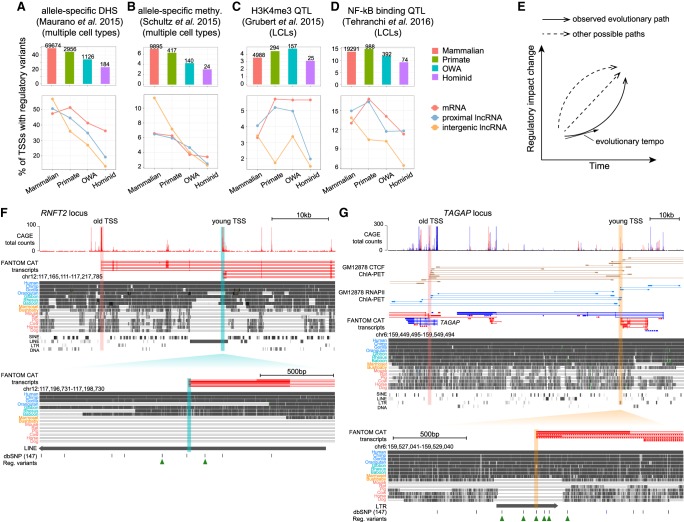
Temporal and spatial constraints on the regulatory evolution of young TSSs. (*A*) (*Top*) Proportion of TSSs harboring regulatory variants associated with allele-specific DHS within TSS ± 1 kb for each TSS group; numbers *above* bars indicate the numbers of TSSs with regulatory variants. (*Bottom*) Proportions of TSSs harboring regulatory variants in different TSS subgroups, defined by transcript type. (*B*) Proportion of TSSs harboring variants associated with allele-specific methylation within TSS ± 1 kb. (*C*) Proportion of TSSs harboring H3K4me3 QTLs within TSS ± 1 kb. Data generated from lymphoblastoid cell lines (LCLs). (*D*) Proportion of TSSs harboring NF-kb complex binding (RELA ChIP) QTLs within TSS ± 1 kb. (*E*) Schematic illustration depicting different possible paths for regulatory evolution of young TSSs. (*F*) Genome browser view of a young TSS *cis*-proximal to old TSSs. (*Top*) FANTOM CAT transcript models (red for forward-strand, blue for reverse-strand); genomic alignments and TE annotations obtained from the UCSC Genome Browser. (*Bottom*) Enlarged region of an “OWA” TSS inside a LINE element. Beneath the alignments are the common SNPs (allele frequency ≥0.01) from dbSNP database and SNPs associated with regulatory variation. (*G*) A young TSS *trans*-proximal to old TSSs. (*Top*) Similar to *F* but with additional CTCF and RNAP II ChIA-PET data for GM12878 cell line. (*Bottom*) Enlarged region of the young TSS. *Below* the alignments are the common SNPs (allele frequency ≥0.01) and regulatory variants.

In our analysis, we examined only *cis*-regulatory variants around TSS loci, as published *trans*-regulatory variants are rare and of relatively low quality. Since the density of *cis*-regulatory variants drops rapidly with increasing distances (The GTEx Consortium 2015), we restricted our analysis to those within ±1 kb of TSSs. By re-analyzing multiple independent data sets, including DHS, methylation, histone marks, and TF binding, we found that young TSSs tend to have fewer regulatory variants compared with old TSSs (see Fig. 5A–D for four representative data sets and Supplemental Fig. S20 for more data sets). This trend is especially clear for variants associated with DHS, methylation, and TF binding. This is interesting because it suggests that, although young TSS loci evolve rapidly, many of the sequence changes appear to have no or limited impact on transcriptional output. To avoid double-counting regulatory variants around closely spaced TSSs, we repeated the analysis on TSSs separated by ≥2 kb which revealed similar trends (though it is possible for a variant to affect multiple adjacent TSSs) (Supplemental Fig. S21). Moreover, trends are maintained when including only regulatory variants with high derived allele frequencies (Supplemental Fig. S22), changes which are more likely to be fixed in populations in the future. Overall, these observations imply that regulatory evolution of young TSSs is subject to a temporal constraint—younger TSSs have a slower tempo in regulatory evolution (Fig. 5E), which might be due to the strong transcriptional repression in early periods.

Separating similarly aged TSSs according to transcript type, mRNA and proximal lncRNA TSSs tend to have more regulatory variants compared with intergenic lncRNA TSSs (Fig. 5A–D). Since mRNA and proximal lncRNA TSSs also have more ChIA-PET interactions than other TSSs (Fig. 4L), we propose that there is a spatial constraint on the regulatory evolution of young TSSs. Generally, younger TSSs have less connectivity to other regulatory modules (i.e., spatially isolated) than older TSSs (Fig. 4E), which likely limits their functional impact. In their subsequent evolution, sequence changes in young TSSs proximal to other regulatory modules tend to have more regulatory effects, and these TSSs may be incorporated in the existing regulatory network more quickly (i.e., at a higher tempo of regulatory evolution). In contrast, relatively isolated TSSs tend to have a slower evolutionary tempo and are more difficult to be co-opted by the host.

Examples of evolving *cis*-proximal (proximal to old TSSs in *cis*) and *trans*-proximal (proximal to old TSSs in *trans* due to chromatin interactions) young TSSs are shown in Figure 5, F and G. In the gene *RNFT2* locus shown in Figure 5F, an “OWA” TSS, which lies on the antisense strand of a newly inserted L1 element, is *cis*-proximal to an upstream old TSS. In surrounding regions of the “OWA” TSS, there are multiple polymorphic sites in current populations, two of which are regulatory variants affecting PU.1 binding and H3K4me3, respectively (Fig. 5F). In the example shown in Figure 5G, a “primate” TSS within an LTR element is ∼70 kb away from the *TAGAP* locus. However, this young TSS is *trans*-proximal to the TSSs of *TAGAP*, as supported by several CTCF and RNAPII ChIA-PET interaction pairs (Fig. 5G). This is a solitary LTR and thus lacks capacity for transposition. Six regulatory variants are within ±1 kb of the young TSS (Fig. 5G). More examples are given in Supplemental Figure S23.

## Discussion

**Figure 6.**
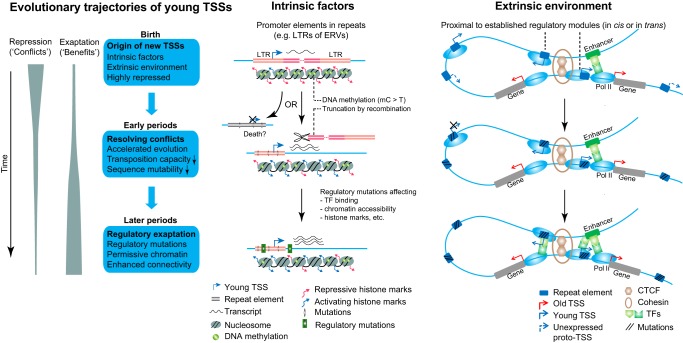
Proposed evolutionary model of young TSSs. The origin of new TSSs is promoted by sequence-intrinsic and -extrinsic factors. In the early phase, newly emerged TSSs undergo rapid sequence evolution, allowing genomic conflicts associated with repeats to be resolved. In the later phases, surviving TSSs gradually gain mutations in surrounding regions which could increase their regulatory capacity.

Given the large number of identified TSSs in the mammalian genomes and the high TSS turnover rate, it is important to understand where new TSSs come from, how they evolve over time, and their functional impact on transcription. Our evolutionary and functional analyses reveal several evolutionary trends: (1) New TSSs tend to have weaker transcription than old ones; (2) they tend to appear in repeat elements and associate with transcripts of uncertain functional status; (3) they are less likely to have a clear regulatory role, as demonstrated by the weaker regulatory potential from functional genomic data; (4) they also tend to appear in already active chromatin regions (e.g., near existing TSSs); and (5) new TSSs evolve more rapidly during their early phase of existence, which may be explained by the inherent instability of neighboring sequences or lack of a vital function. We summarize our main findings in an integrative model as shown in Figure 6.

First, our analyses revealed several intrinsic and extrinsic factors that promote the emergence of new TSSs (Fig. 6). Intrinsic factors are mainly associated with repeats, among which retrotransposons are a major contributor. For LTR-associated TSSs, the transcription initiation seems mainly driven by the original promoter activities within LTRs (Fig. 2; Supplemental Fig. S5). For TSSs associated with non-LTR transposons, the underlying mechanisms are less clear. The antisense promoter of L1 is well-documented (Speek 2001), so TSSs at these locations (Fig. 2C) may have initiated transcription soon after transposon insertion without any new mutations. Other transposon-associated TSSs could be due to as yet unknown cryptic promoter structures that existed in newly inserted transposons, or require mutations to enable transcription initiation. Genome organization and chromatin environment are likely important extrinsic factors. New TSSs are usually proximal in *cis* or *trans* to other established transcriptional units providing easier access to the transcriptional machinery, whereas unexpressed proto-TSSs are more isolated. This dependence on extrinsic chromatin environments partly explains why only a small fraction of proto-TSSs have detectable initiation signals.

Second, resolving genomic conflicts is a likely major theme in the early phase of young TSS existence (Fig. 6). Our evolutionary rate analysis revealed that young TSSs experience rapid sequence evolution at first, which appears to be associated with several endogenous mutational processes, including DNA methylation, recombination, and tandem repeat mutagenesis. We suggest that such rapid evolution can reduce genomic conflicts caused by the instability of repeats associated with young TSSs, as the TSS loci became more stable after they mutated. We suspect that a considerable fraction of new TSSs may die out during this early phase of rapid evolution.

Third, although new TSSs tend to have limited transcriptional competence in the beginning, the regulatory potential of surviving young TSSs appears to be gradually enhanced in later periods (Fig. 6). By examining regulatory variants around TSS loci, we revealed that the evolution of regulatory functions of young TSSs appears to be subject to temporal and spatial constraints. The temporal constraint—that younger TSSs have fewer regulatory variants within a period (slower tempo) despite faster sequence evolution—is probably due to the genomic conflicts caused by instability of associated repeats and novel transcripts of uncertain functional status. The spatial constraint—that TSSs with fewer chromosomal contacts display a slower tempo of regulatory evolution—likely limits the regulatory impact of young TSSs and affects the evolutionary trajectories of young TSSs in different contexts. We speculate that among younger TSSs, more isolated ones are more likely to die out during evolution.

Many studies have reported the contribution of repetitive sequences to regulatory innovation (Gerdes et al. 2016; Chuong et al. 2017). For example, LTR-derived regulatory elements have been reported in placenta (Emera and Wagner 2012; Chuong et al. 2013), pluripotency and embryonic development (Fort et al. 2014; Grow et al. 2015; Durruthy-Durruthy et al. 2016), and immune systems (Chuong et al. 2016; Manghera et al. 2016). A recent H3K27ac and H3K4me1 ChIP-seq study profiling *cis*-regulatory elements suggests that transposable elements are the primary source of gene regulatory innovation in primates (Trizzino et al. 2017). Our work neatly complements findings from these studies. We have shown that repeat-derived TSSs are tightly constrained in the beginning and have limited functional impact, but after resolving genomic conflicts, some are successfully incorporated into the existing regulatory network, turning “conflicts” into “benefits” (Chuong et al. 2017). In the long run, the repeat-derived TSSs contribute significantly to regulatory innovation. A similar evolutionary pattern was also observed in *Alu* exonization in primate genomes (Attig et al. 2016). Given the pervasiveness of repetitive sequences and the similarity of chromatin structures in eukaryotic genomes, the observed evolutionary processes in primate TSSs could also exist in other eukaryotic groups. These evolutionary patterns also highlight the genomic robustness and evolvability in regulatory evolution (Wagner 2007).

Several aspects of the current study may be improved in the future. First, as RepeatMasker annotations rely on sequence similarity of repeat elements with their consensus sequences, highly diverged repeats (especially old repeats) could be missed. A previous study suggests that repeat elements may comprise over two-thirds of the human genome (de Koning et al. 2011), so further improvement of repeat annotations would be beneficial. Second, we assessed the functional impact of the sequence changes around the TSSs using regulatory variants associated with relevant molecular traits (e.g., DHS); however, if CAGE-defined TSSs for human populations were to become available, like that for *Drosophila* (Schor et al. 2017), it would be possible to identify so-called “tssQTLs” that more accurately measure the transcriptional impact of sequence changes. Third, we note that the fewer regulatory variants detected in younger or more isolated TSSs may partly reflect the reduced statistical power owing to the lower read-counts of functional assays in those regions, thus further assessment with improved data sets could refine our analysis. Fourth, many young TSSs are associated with lncRNAs, but a general understanding of lncRNAs remains limited. Further detailed characterization of lncRNAs will help to elucidate their functional roles and underlying evolutionary forces. Finally, our definition of TSS ages is based on sequence, rather than functional, homology, and further high-quality TSS data in other primate species should facilitate in-depth comparative analyses and deepen our understanding of human TSSs.

## Methods

Detailed methods are provided in Supplemental Methods. Supplemental Table S5 provides URL links for publicly available data sets used in this study. A tab-delimited spreadsheet containing the defined TSS groups/subgroups and other related data generated in this study is provided in Supplemental Table S6.

### Human TSS annotation data set

The “robust” TSS annotation from the latest FANTOM CAT annotation (part of FANTOM5; dated Aug. 9, 2017) was used (Hon et al. 2017). Unless specified otherwise, we used the dominant TSS position (the most frequently used initiation site) for each TSS peak provided by FANTOM.

### Grouping human TSSs by sequence age

To estimate the sequence ages of human TSSs, the UCSC liftOver tool (Tyner et al. 2017) was used to determine presence or absence of each human TSS sequence in other nonhuman genomes based on available pairwise chain alignment files. A human TSS locus was considered present in another genome if the corresponding pairwise alignment satisfies (1) a mapping ratio of the human TSS peak (i.e., a CAGE tag cluster region predicted by FANTOM) in another genome of ≥90%, and (2) a mapping ratio of the TSS peak ±100 bp (considered as the core promoter region in this study) of ≥50%. We carefully filtered TSSs that could confound downstream analyses. See Supplemental Methods for details.

### Analysis of TATA-box and CpG islands

A TSS was considered CGI-associated if it overlapped a CGI (TSS ± 100 bp); CGI annotations were obtained from Cohen et al. (2011). A TSS was considered TATA-box-associated if the start of the TATA-box motif was located 25–35 bp upstream of the TSS; TATA-box hits were predicted using the TBP position-weighted matrix from the JASPAR database (Mathelier et al. 2016) with a minimum score of 80%.

### Analysis of repeats associated with TSSs

A TSS was considered associated to a repeat (TEs or tandem repeats) if it overlapped an element within TSS ± 100 bp, taking the nearest one to the TSS if there are multiple overlapping repeat elements. Repeat annotations were obtained from RepeatMasker (Tarailo-Graovac and Chen 2009), TRF (Benson 1999), and STRcat (Willems et al. 2014). For most downstream analyses, we focused on the nearest retrotransposons and grouped the TSSs into four categories (“SINE,” “LINE,” “LTR,” and “Others”). Statistics for TSSs associated with TEs are given in Supplemental Table S4. More details are given in Supplemental Methods.

To analyze the distributions of TSSs along repeat elements, we calculated the relative distances of TSSs to the 5′ end of the corresponding consensus repeat sequences based on alignments provided by RepeatMasker.

### Evolutionary rate analysis

To study evolutionary rates, we extracted multiple genomic alignments of TSS loci (TSS ± 1 kb) from UCSC (Tyner et al. 2017). We re-aligned the extracted alignments using PRANK to improve the alignment quality (Loytynoja and Goldman 2008) and inferred ancestral sequence changes for each TSS locus using FastML (Ashkenazy et al. 2012). More details are given in Supplemental Methods.

### Analysis of mutational mechanisms

We used the published germline methylation data from Guo et al. (2015) and focused on CpG methylation events. The methylome of male primordial germ cells of 7-wk-old embryos was used because this sample exhibited a high level of methylation across the genome, although we obtain analogous results with all samples. Data of recombination rates in human populations were obtained from the HapMap project (The International HapMap Consortium 2007). The completeness status (solitary or nonsolitary) of LTRs was predicted by REANNOTATE (Pereira 2008) with parameters “-n –c,” using the RepeatMasker annotation as input.

### Analysis of functional signatures of TSSs

Processed ENCODE data for DHS, ChIP-seq, and DNA methylation of the GM12878, K562, and H1-hESC cell lines were downloaded from ENCODE (Rosenbloom et al. 2013) and Ensembl (Aken et al. 2017). Analysis and visualization of functional genomic data were performed with BEDTools (Quinlan 2014), R (R CoreTeam 2015), SeqPlots (Stempor and Ahringer 2016), and deepTools (Ramirez et al. 2014).

CTCF and RNAPII ChIA-PET data in the GM12878 cell line were obtained from Tang et al. (2015), RAD21 ChIA-PET data in GM12878 from Grubert et al. (2015), and RNAPII ChIA-PET data in K562 from ENCODE.

### Regulatory variant analysis

Regulatory variants were obtained from Grubert et al. (2015), Maurano et al. (2015), Schultz et al. (2015), and Tehranchi et al. (2016). The derived allele frequencies (DAFs) of variants were obtained from the 1000 Genomes Project Phase 3 release (The 1000 Genomes Project Consortium 2012), and only variants with known ancestral alleles were used. For each type of regulatory variant, we calculated the proportion of TSSs harboring at least one regulatory variant with ±1 kb. To account for possible double-counting of variants from adjacent TSS loci, we repeated the analysis after excluding TSSs separated by <2 kb. We also repeated the analysis using three different minimum DAF thresholds (0.01, 0.1, and 0.5).

## Data access

All the analyses in this study were based on published data sets. A tab-delimited spreadsheet containing the defined TSS groups/subgroups and other related data generated in this study is provided in Supplemental Table S6.

## Supplementary Material

Supplemental Material
