# Supplementary material for: Integrated analysis sheds light on evolutionary trajectories of young transcription start sites in the human genome
Source: Genome Res. 2018 May;28(5):676–88. doi: 10.1101/gr.231449.117 (PMC5932608; doi:10.1101/gr.231449.117)
Supplement: Supplemental Material [file supp_28_5_676__index.html]

Integrated analysis sheds light on evolutionary trajectories of young transcription start sites in the human genome — Supplemental Material 

# Integrated analysis sheds light on evolutionary trajectories of young transcription start sites in the human genome

## Supplemental Material

- Supplemental\_Table\_S1.docx
- Supplemental\_Table\_S2.docx
- Supplemental\_Table\_S3.docx
- Supplemental\_Table\_S4.docx
- Supplemental\_Table\_S5.docx
- Supplemental\_Methods.docx
- Supplemental\_Fig\_S1.pdf
- Supplemental\_Fig\_S2.pdf
- Supplemental\_Fig\_S3.pdf
- Supplemental\_Fig\_S4.pdf
- Supplemental\_Fig\_S5.pdf
- Supplemental\_Fig\_S6.pdf
- Supplemental\_Fig\_S7.pdf
- Supplemental\_Fig\_S8.pdf
- Supplemental\_Fig\_S9.pdf
- Supplemental\_Fig\_S10.pdf
- Supplemental\_Fig\_S11.pdf
- Supplemental\_Fig\_S12.pdf
- Supplemental\_Fig\_S13.pdf
- Supplemental\_Fig\_S14.pdf
- Supplemental\_Fig\_S15.pdf
- Supplemental\_Fig\_S16.pdf
- Supplemental\_Fig\_S17.pdf
- Supplemental\_Fig\_S18.pdf
- Supplemental\_Fig\_S19.pdf
- Supplemental\_Fig\_S20.pdf
- Supplemental\_Fig\_S21.pdf
- Supplemental\_Fig\_S22.pdf
- Supplemental\_Fig\_S23.pdf
- Supplemental\_Table\_S6.zip
