# Supplementary material for: Integrated analysis sheds light on evolutionary trajectories of young transcription start sites in the human genome
Source: Genome Res. 2018 May;28(5):676–88. doi: 10.1101/gr.231449.117 (PMC5932608; doi:10.1101/gr.231449.117)
Supplement: Supplemental Material [file supp_gr.231449.117_Supplemental_Table_S1.docx]

Supplemental Table S1 Species and genome assemblies used for estimating sequence ages of TSSs.

| **Species** | **Assembly version** | **Taxa** | | | |
| --- | --- | --- | --- | --- | --- |
| Human | hg19 | Hominids | Old World anthropoids | Primates | Mammals |
| Chimp | panTro4 |  |  |  |  |
| Gorilla | gorGor3 |  |  |  |  |
| Orangutan | ponAbe2 |  |  |  |  |
| Gibbon | nomLeu3 |  |  |  |  |
| Rhesus | rheMac3 |  |  |  |  |
| Baboon | papHam1 |  |  |  |  |
| Marmoset | calJac3 |  |  |  |  |
| Tarsier | tarSyr1 |  |  |  |  |
| Mouse lemur | micMur1 |  |  |  |  |
| Bushbaby | otoGar1 |  |  |  |  |
| Mouse | mm10 |  |  |  |  |
| Rat | rn6 |  |  |  |  |
| Pig | susScr3 |  |  |  |  |
| Cow | bosTau7 |  |  |  |  |
| Horse | equCab2 |  |  |  |  |
| Dog | canFam3 |  |  |  |  |
