# Supplementary material for: Integrated analysis sheds light on evolutionary trajectories of young transcription start sites in the human genome
Source: Genome Res. 2018 May;28(5):676–88. doi: 10.1101/gr.231449.117 (PMC5932608; doi:10.1101/gr.231449.117)
Supplement: Supplemental Material [file supp_gr.231449.117_Supplemental_Table_S2.docx]

Supplemental Table S2 Statistics of grouping results with different sets of cutoffs for liftOver, after filtering the TSSs overlapping blacklist regions.

| **Min mapped % of TSS peaks** | **Min mapped % of TSS peak±100 bp** | **Min chain size** | **Mammalian** | **Primate** | **OWA** | **Hominid** | **Used in final analyses?** |
| --- | --- | --- | --- | --- | --- | --- | --- |
| 0.9 | 0.5 | 10kb | 141,117 | 6,668 | 3,318 | 799 | Yes |
| 0.8 | 0.5 | 10kb | 142,782 | 5,531 | 2,902 | 687 | No |
| 0.5 | 0.5 | 10kb | 144,121 | 4,652 | 2,532 | 597 | No |
| 0.9 | 0.3 | 10kb | 141,288 | 6,559 | 3,264 | 791 | No |
| 0.9 | 0.7 | 10kb | 139,652 | 7,505 | 3,840 | 905 | No |
| 0.9 | 0.5 | 5kb | 141,328 | 6,716 | 3,109 | 749 | No |
| 0.9 | 0.5 | 20kb | 140,913 | 6,591 | 3,525 | 873 | No |
