# Supplementary material for: Integrated analysis sheds light on evolutionary trajectories of young transcription start sites in the human genome
Source: Genome Res. 2018 May;28(5):676–88. doi: 10.1101/gr.231449.117 (PMC5932608; doi:10.1101/gr.231449.117)
Supplement: Supplemental Material [file supp_gr.231449.117_Supplemental_Table_S3.docx]

Supplemental Table S3 Lists of blacklist genomic regions used for filtering TSSs.

| **File** | **Source** |
| --- | --- |
| wgEncodeDukeMapabilityRegionsExcludable.bed | ENCODE |
| wgEncodeDacMapabilityConsensusExcludable.bed | ENCODE |
| seq.cov1.ONHG19.bed | Pickrell et al. 2011 |
| UM1K0M50BP.bed | Li and Freudenberg 2014 |
