# Supplementary material for: Integrated analysis sheds light on evolutionary trajectories of young transcription start sites in the human genome
Source: Genome Res. 2018 May;28(5):676–88. doi: 10.1101/gr.231449.117 (PMC5932608; doi:10.1101/gr.231449.117)
Supplement: Supplemental Material [file supp_gr.231449.117_Supplemental_Table_S4.docx]

Supplemental Table S4 Statistics of TSS subgroups defined by transcript types and the nearest retrotransposon elements.

| **Mammalian** | | | **Primate** | | | **Old World Anthropoid** | | | **Hominid** | | |
| --- | --- | --- | --- | --- | --- | --- | --- | --- | --- | --- | --- |
| mRNA | SINE | 3427 | mRNA | SINE | 271 | mRNA | SINE | 100 | mRNA | SINE | 16 |
|  | LINE | 3470 |  | LINE | 299 |  | LINE | 118 |  | LINE | 43 |
|  | LTR | 830 |  | LTR | 433 |  | LTR | 306 |  | LTR | 61 |
|  | Others | 75301 |  | Others | 1569 |  | Others | 555 |  | Others | 145 |
| proximal lncRNA | SINE | 2019 | proximal lncRNA | SINE | 204 | proximal lncRNA | SINE | 89 | proximal lncRNA | SINE | 15 |
|  | LINE | 2311 |  | LINE | 266 |  | LINE | 164 |  | LINE | 34 |
|  | LTR | 827 |  | LTR | 465 |  | LTR | 309 |  | LTR | 67 |
|  | Others | 35202 |  | Others | 1071 |  | Others | 426 |  | Others | 87 |
| intergenic lncRNA | SINE | 966 | intergenic lncRNA | SINE | 84 | intergenic lncRNA | SINE | 43 | intergenic lncRNA | SINE | 8 |
|  | LINE | 1232 |  | LINE | 219 |  | LINE | 173 |  | LINE | 53 |
|  | LTR | 1192 |  | LTR | 799 |  | LTR | 524 |  | LTR | 146 |
|  | Others | 9106 |  | Others | 516 |  | Others | 269 |  | Others | 58 |
| other RNA | SINE | 324 | other RNA | SINE | 32 | other RNA | SINE | 11 | other RNA | SINE | 0 |
|  | LINE | 368 |  | LINE | 78 |  | LINE | 39 |  | LINE | 17 |
|  | LTR | 147 |  | LTR | 99 |  | LTR | 67 |  | LTR | 17 |
|  | Others | 4395 |  | Others | 263 |  | Others | 125 |  | Others | 32 |
