# Supplementary material for: Integrated analysis sheds light on evolutionary trajectories of young transcription start sites in the human genome
Source: Genome Res. 2018 May;28(5):676–88. doi: 10.1101/gr.231449.117 (PMC5932608; doi:10.1101/gr.231449.117)
Supplement: Supplemental Material [file supp_gr.231449.117_Supplemental_Table_S5.docx]

Supplemental Table S5 URL links of published datasets used in this study.

| **Data** | **Source links** |
| --- | --- |
| FANTOM TSSs | <http://fantom.gsc.riken.jp/5/suppl/Hon_et_al_2016/data/> (human)  <http://fantom.gsc.riken.jp/5/datafiles/latest/extra/CAGE_peaks/> (non-human) |
| liftOver chain files | <http://hgdownload.cse.ucsc.edu/goldenPath/hg19/liftOver/> |
| RepeatMasker annotation | <http://www.repeatmasker.org/genomes/hg19/RepeatMasker-rm405-db20140131/hg19.fa.out.gz> |
| TRF | <http://hgdownload.cse.ucsc.edu/goldenpath/hg19/database/simpleRepeat.txt.gz> |
| STRcat | <http://strcat.teamerlich.org/download> |
| MULTIZ alignments | <http://hgdownload.cse.ucsc.edu/goldenPath/hg19/multiz100way/maf/> |
| Germline methylation | <https://www.ncbi.nlm.nih.gov/geo/query/acc.cgi?acc=GSE63818> |
| Variants from 1000 genomes project | <ftp://ftp.1000genomes.ebi.ac.uk/vol1/ftp/release/20130502/>  <http://hgdownload.cse.ucsc.edu/goldenPath/hg19/encodeDCC/> |
| ENCODE functional datasets | <ftp://ftp.ebi.ac.uk/pub/databases/ensembl/encode/integration_data_jan2011/> |
| ChIA-PET data | <https://www.ncbi.nlm.nih.gov/geo/query/acc.cgi?acc=GSE62742>  <https://www.ncbi.nlm.nih.gov/geo/query/acc.cgi?acc=GSE72816> |
| AS or QTL data | DHS:  <http://www.nature.com/ng/journal/v47/n12/extref/ng.3432-S5.txt>  DNA methylation: <https://www.nature.com/nature/journal/v523/n7559/extref/nature14465-s2.zip>  Histone modifications:  <http://mitra.stanford.edu/kundaje/portal/chromovar3d/index.html>  TF binding:  <http://www.cell.com/cms/attachment/2062331538/2064077614/mmc2.xlsx> |
