# Supplementary material for: Integrated analysis sheds light on evolutionary trajectories of young transcription start sites in the human genome
Source: Genome Res. 2018 May;28(5):676–88. doi: 10.1101/gr.231449.117 (PMC5932608; doi:10.1101/gr.231449.117)
Supplement: Supplemental Material [file supp_gr.231449.117_Supplemental_Methods.docx]

**Defining TSS groups by sequence age**

To categorize human TSSs by the evolutionary age, we made use of whole genome alignments between human (hg19) and 16 other mammalian genomes (**Supplemental Table S1**) from UCSC Genome Browser ([Tyner et al. 2017](#_ENREF_9)). We required a minimum liftOver mapping ratio of 90% for CAGE TSS peaks (~23bp in length on average), which usually covers Initiator (Inr) elements of promoters ([Taylor et al. 2006](#_ENREF_8)). In addition, we required a minimum mapping ratio of 50% for TSS peaks±100 bp, which we considered to be the “core promoter” region in our study and are usually under high selective constraint ([Taylor et al. 2006](#_ENREF_8)), although there is no standard definition for “core promoter” currently. To reduce potential false positives resulting from alignments of paralogous loci in two genomes, we further required a minimum alignment chain size of 10 kb for both target and query genomes. A human TSS locus satisfying the above criteria for the pairwise alignment was considered as having the orthologous sequence in the surveyed genome, and its sequence age should be equal to or larger than the age of last common ancestor of two species. Based upon the presence/absence patterns in alignments, we categorized the human TSSs into four groups of different ages: 1) TSSs whose sequence loci can be found in at least one non-primate mammalian genome, named ‘mammalian’ group; 2) TSSs whose sequences occurred during early primate evolution but before the last common ancestor of Old World anthropoids, named ‘primate’ group; 3) TSSs whose sequences occurred during the evolution of Old World anthropoids but before the last common ancestor of hominids, named ‘OWA’ group; 4) TSSs whose sequences occurred since emergence of hominids, named ‘hominid’ group.

We also tested multiple sets of liftOver thresholds which did not result in notable variation in the grouping results (**Supplemental Table S2**). We checked whether sequence alignments could be used as a suitable proxy for presence/absence of TSSs using FANTOM CAGE-defined TSSs (‘robust’ annotations) of four other mammalian species (macaque, mouse, rat and dog). Since the human genome has the most CAGE annotations, we assessed presence/absence patterns of non-human TSS loci in the human genome (using the same alignment criteria as above) and checked how many mapped loci also have a CAGE-defined TSS (**Supplemental Fig. S1**).

**Definition of transcription types**

Coding status and transcript classification were obtained from the FANTOM CAT annotation (part of FANTOM5). To facilitate analysis and interpretation, we merged three lncRNA classes (“lncRNA_antisense”, “lncRNA_divergent” and “lncRNA_sense_intronic”) into a class called “proximal lncRNA”. We also merged several minor classes (“sense_overlap_RNA”, “short_ncRNA”, “small_RNA”, “structural_RNA” and “uncertain_coding”) into a class called “other RNA”. For TSSs which are associated with multiple types of transcripts, we assigned them hierarchically to the five categories: mRNA > proximal_lncRNA > intergenic_lncRNA > pseudogene > other_RNA.

**Filtering TSSs that could confound downstream analyses**

As highly repetitive genomic regions could lead to poor assemblies and erroneous alignments, we filtered out any TSS whose ±1 kb regions overlapping the blacklisted genomic regions (see **Supplemental Table S3**) defined by ENCODE and two other studies ([Pickrell et al. 2011](#_ENREF_6); [Li and Freudenberg 2014](#_ENREF_4)). Because CAGE reads are usually short (20~70bp) ([Kanamori-Katayama et al. 2011](#_ENREF_3)) and can be mapped to the genome multiple times, we made use of the Duke 20-bp uniqueness track from UCSC Genome Browser to filter out TSS peaks that have an average uniqueness score of <0.5 (a 20-bp uniqueness score of <0.5 means that a 20-mer can be mapped to the human genome more than twice). Some TSS loci still exhibited suspiciously high read depths in some functional genomic datasets, suggesting artifacts due to poor mappability in low-complexity tandem repeat regions; therefore we further filtered any TSS harboring >10% (i.e., >200 bp) tandem repeats within ±2kb. In addition, TSSs on chrM and chrY were excluded from all analyses, as some genome assemblies or functional datasets lack data for these chromosomes.

We identified two further significant sources of potential false positives in the FANTOM annotation. First are pseudogene-associated TSSs (annotated by FANTOM CAT); pseudogenes were reported as a notable source of false positives for CAGE-defined TSSs because of their high sequence similarity to the original gene loci and the short lengths of CAGE reads ([Zhao et al. 2011](#_ENREF_11)). For the GM12878 cell line, only 3.7% of primate lineage TSSs associated with pseudogenes have a corresponding transcriptional signal in GRO-cap-defined TSSs (**Supplemental Fig. S8**) ([Core et al. 2014](#_ENREF_2)), suggesting that most such TSSs do not have any detectable transcription (For comparison, 43% of TSSs that are not associated with pseudogenes or poly(dA:dT) tracts can be found in the GRO-cap-defined TSS dataset). Therefore, we excluded all pseudogene TSSs from downstream analyses. Second are TSSs associated with poly(A) or poly(T) tracts. In the GM12878 cell line, only 5.2% of poly(dA:dT)-associated TSSs in the primate lineage can be found among the GRO-cap-defined TSSs (**Supplemental Fig. S8**). Therefore we filtered out the TSSs flanked by a tandem repeat with A content of >50 % or T content of >50 % within ±100bp.

**Analysis of repeats associated with TSSs**

The annotation of transposable elements in our analysis was based on the RepeatMasker annotations for the hg19 assembly (Repeat Library 20140131) ([Tarailo-Graovac and Chen 2009](#_ENREF_7)). As young TSS loci are frequently associated with tandem repeats, tandem repeats annotated by TRF (downloaded from UCSC) and STRcat ([Willems et al. 2014](#_ENREF_10)) were also used. The “Simple repeat”, “Low complexity” and “Satellite” families in RepeatMasker were considered as tandem repeats here and merged with the TRF and STRcat. For overlapping tandem repeats in these three datasets, the priority order for inclusion was STRcat > TRF > RepeatMasker.

To investigate the repeat content around TSS loci, we first identified the nearest repeat element to each TSS and counted how many TSSs harbored repeat elements within TSS±100 bp regions. Since retrotransposons and tandem repeats were the main types of TSS-associated repeats and many tandem repeats were derived from retrotransposons, for each TSS group defined by sequence age, we further defined four TSS subgroups (‘SINE-associated’, ‘LINE-associated’, ‘LTR-associated’ and ‘Others’) based on the nearest retrotransposon within 100 bp of the TSS.

To see whether specific types of retrotransposons are over-represented in a specific TSS group, we simulated 200,000 random intervals in the human genome (based on the same length distribution as that of FANTOM TSS peak lengths; simulation was performed using the BEDtools ‘shuffle’ command), and applied the same classification method to simulated intervals to determine four age groups as that for TSSs. We identified the nearest repeat elements of simulated intervals in each age group and compared the composition of repeat classes in simulated intervals to that of FANTOM TSS peaks.

**Distances of young TSSs to CTCF or RAD21 interaction loci**

When investigating distances of young TSSs to ChIA-PET interaction loci of CTCF or RAD21, we only considered the mammalian-conserved interaction pairs, i.e., whose sequences could be found in at least one of the six non-primate mammalian genomes listed in **Supplemental Table S1**, based on the liftOver mapping with parameters “-minMatch=0.5 -minChainT=10000 -minChainQ=10000”.

**Evolutionary rate analysis**

We investigated the sequence evolutionary rates around TSS loci for two recent periods (from the last common ancestor of OWAs to the last common ancestor of hominids and from the last common ancestor of hominids to present). We extracted multiple alignments for all human TSS±1kb regions against 14 other mammalian genomes from the 100-way MULTIZ genome alignments provided by UCSC (all species used for analysis are listed in **Supplemental Table S1**; tarSyr1 and micMur1 were unavailable and so were excluded from this analysis). To improve alignment quality, the extracted MULTIZ alignments were re-aligned using PRANK with parameter “+F”, which was found to generate more accurate gapped alignments for evolutionary analysis ([Loytynoja and Goldman 2008](#_ENREF_5)). The re-alignment results were then used to infer ancestral sequences for each TSS locus using FASTML with parameters “--SubMatrix HKY -jointReconstruction no --indelReconstruction ML” ([Ashkenazy et al. 2012](#_ENREF_1)). FASTML produced posterior probabilities for each position of inferred ancestral sequences. Positions with low-confidence inferred sequences (maximum marginal probability of <0.8) were excluded for subsequent analyses. Evolutionary sequence changes (substitutions, insertions and deletions) in TSS loci in different periods were identified by comparing inferred ancestral sequences and derived sequences; these changes were used to calculate substitution, insertion, and deletion rates for each period respectively. To estimate the genomic average evolutionary rates, we generated 10,000 random 2-kb intervals from the human genome, and ran the same analysis pipeline as described above for the TSS loci. The relative rates of substitutions, insertions and deletions in TSS loci were then obtained by dividing the original rates by genomic average rates estimated from random intervals.

**References**

Ashkenazy H, Penn O, Doron-Faigenboim A, Cohen O, Cannarozzi G, Zomer O, Pupko T. 2012. FastML: a web server for probabilistic reconstruction of ancestral sequences. *Nucleic acids research* **40**(Web Server issue): W580-584.

Core LJ, Martins AL, Danko CG, Waters CT, Siepel A, Lis JT. 2014. Analysis of nascent RNA identifies a unified architecture of initiation regions at mammalian promoters and enhancers. *Nature genetics* **46**(12): 1311-1320.

Kanamori-Katayama M, Itoh M, Kawaji H, Lassmann T, Katayama S, Kojima M, Bertin N, Kaiho A, Ninomiya N, Daub CO et al. 2011. Unamplified cap analysis of gene expression on a single-molecule sequencer. *Genome research* **21**(7): 1150-1159.

Li W, Freudenberg J. 2014. Characterizing regions in the human genome unmappable by next-generation-sequencing at the read length of 1000 bases. *Computational biology and chemistry* **53 Pt A**: 108-117.

Loytynoja A, Goldman N. 2008. Phylogeny-aware gap placement prevents errors in sequence alignment and evolutionary analysis. *Science* **320**(5883): 1632-1635.

Pickrell JK, Gaffney DJ, Gilad Y, Pritchard JK. 2011. False positive peaks in ChIP-seq and other sequencing-based functional assays caused by unannotated high copy number regions. *Bioinformatics* **27**(15): 2144-2146.

Tarailo-Graovac M, Chen N. 2009. Using RepeatMasker to identify repetitive elements in genomic sequences. *Current protocols in bioinformatics* **Chapter 4**: Unit 4 10.

Taylor MS, Kai C, Kawai J, Carninci P, Hayashizaki Y, Semple CA. 2006. Heterotachy in mammalian promoter evolution. *PLoS genetics* **2**(4): e30.

Tyner C, Barber GP, Casper J, Clawson H, Diekhans M, Eisenhart C, Fischer CM, Gibson D, Gonzalez JN, Guruvadoo L et al. 2017. The UCSC Genome Browser database: 2017 update. *Nucleic acids research* **45**(D1): D626-D634.

Willems T, Gymrek M, Highnam G, Genomes Project C, Mittelman D, Erlich Y. 2014. The landscape of human STR variation. *Genome research* **24**(11): 1894-1904.

Zhao X, Valen E, Parker BJ, Sandelin A. 2011. Systematic clustering of transcription start site landscapes. *PloS one* **6**(8): e23409.
