# Supplementary material for: Integrated analysis sheds light on evolutionary trajectories of young transcription start sites in the human genome
Source: Genome Res. 2018 May;28(5):676–88. doi: 10.1101/gr.231449.117 (PMC5932608; doi:10.1101/gr.231449.117)
Supplement: Supplemental Material [file supp_gr.231449.117_Supplemental_Fig_S1.pdf]

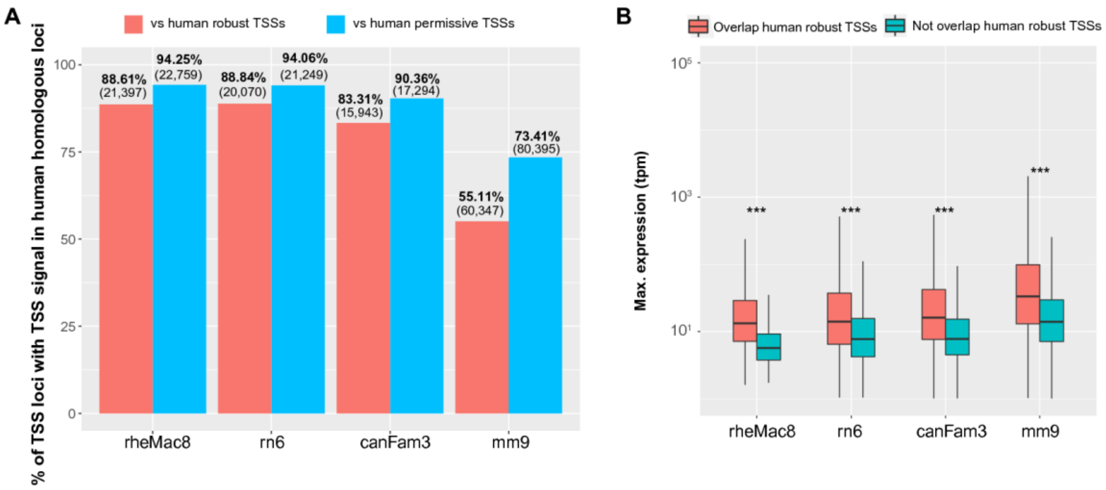

Supplemental Figure S1 Comparing CAGE-defined TSSs of human and other species in alignable regions (**A**) Assessing how many CAGE-defined non-human TSSs ('robust' annotations) that can be aligned to the human genome have CAGE-defined TSSs within the corresponding loci (mapped TSS locus±100bp, same transcriptional strand) in human (compared to human 'robust' and 'permissive' annotations respectively). Inside the brackets are numbers of mapped TSSs between two species. (**B**) Comparing the expression levels of the alignable non-human TSSs with robust CAGE signal in human homologous loci to those without robust CAGE signal in human. "Max. expression" means the maximum expression level across all samples for each TSS, using the expression data from FANTOM. Statistical significance was calculated using the Wilcoxon rank sum test ("***", $p < 0.001$). For those non-human TSSs that can be aligned to human genome but don't have CAGE-defined TSSs in human, it could be due to: 1) lack of CAGE-seq data for tissues/cell types/developmental stages expressing such TSSs in human; 2) very low expression in human thus difficult to detect by current techniques; and 3) no expression in human at all (i.e. the "expression turnover without sequence turnover" scenario defined in Young et al. 2015).
