# Supplementary figures and images for: Integrated analysis sheds light on evolutionary trajectories of young transcription start sites in the human genome
Source: Genome Res. 2018 May;28(5):676–88. doi: 10.1101/gr.231449.117 (PMC5932608; doi:10.1101/gr.231449.117)

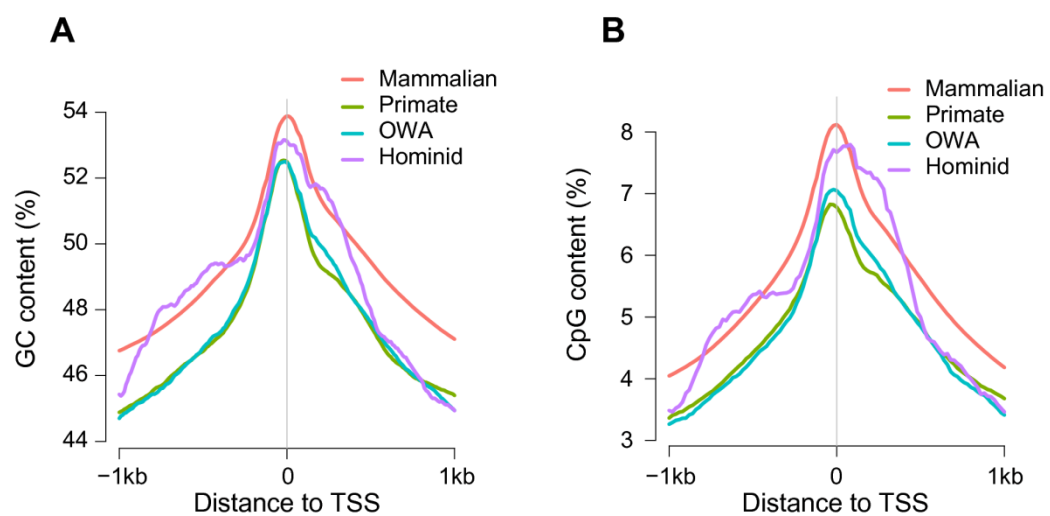

**Supplemental Figure S2 Comparison of GC content (A) and CpG content (B) between four groups.**

Supplement: Supplemental Material [file supp_gr.231449.117_Supplemental_Fig_S2.pdf]
