# Supplementary material for: Integrated analysis sheds light on evolutionary trajectories of young transcription start sites in the human genome
Source: Genome Res. 2018 May;28(5):676–88. doi: 10.1101/gr.231449.117 (PMC5932608; doi:10.1101/gr.231449.117)
Supplement: Supplemental Material [file supp_gr.231449.117_Supplemental_Fig_S3.pdf]

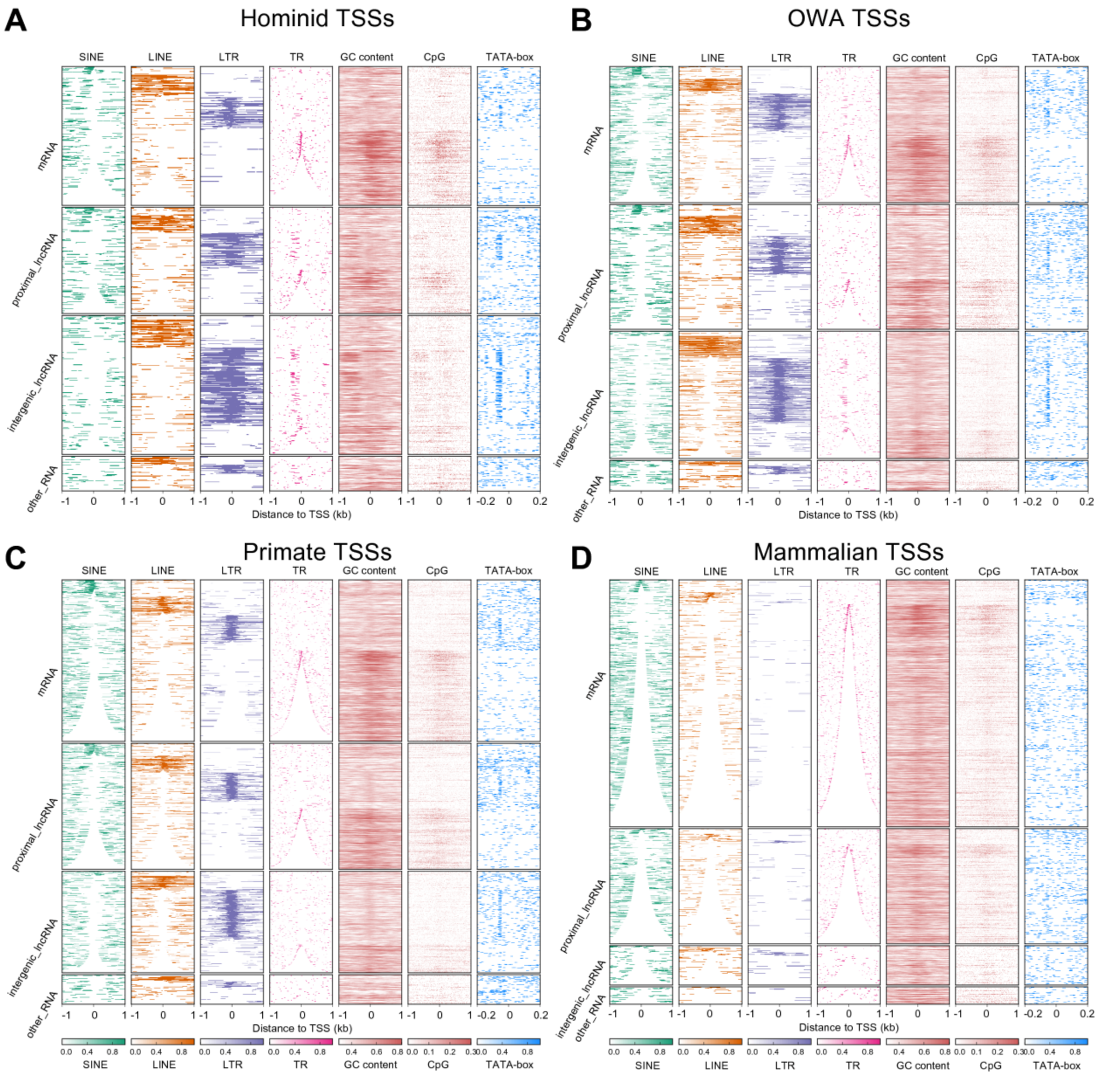

Supplemental Figure S3 Heatmap for repeat content, GC content, CpG content and TATA-box in four TSS groups. Each TSS group is subdivided into subgroups based on transcript type. Within each subgroup, rows are sorted by the distance from the TSS to the nearest TE element (priority order: SINE > LINE > LTR > Others). For the TSSs in the 'Others' category, rows are sorted based on their distances to the nearest tandem repeat elements. The color gradients for repeat elements (SINE, LINE, LTR, tandem repeat (TR)) represent the repeat coverage in 10 bp bins.Regions shown in the TATA-box columns are TSS±200 bp. Because of the large number of TSSs in the 'mammalian' group (panel **D**), only the data of 5000 randomly selected TSSs are shown.
