# Supplementary material for: Integrated analysis sheds light on evolutionary trajectories of young transcription start sites in the human genome
Source: Genome Res. 2018 May;28(5):676–88. doi: 10.1101/gr.231449.117 (PMC5932608; doi:10.1101/gr.231449.117)
Supplement: Supplemental Material [file supp_gr.231449.117_Supplemental_Fig_S4.pdf]

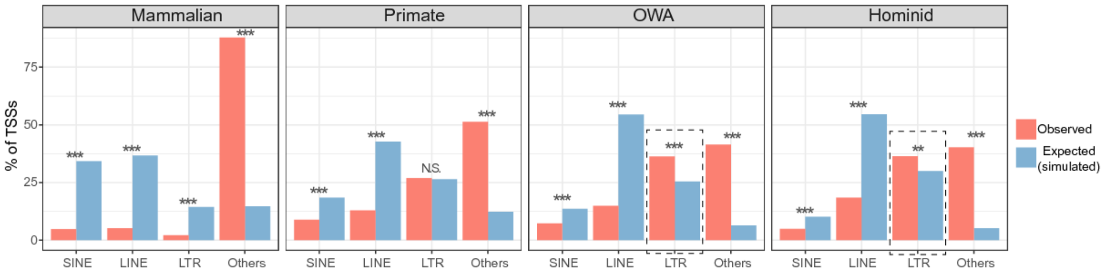

Supplemental Figure S4 Comparison of observed composition of retrotransposons in each age group of TSSs to the expected composition based on random intervals. Statistical significance was calculated using Fisher's exact test ("**", $p < 0.01$; "***", $p < 0.001$; N.S., not significant).
