# Supplementary material for: Integrated analysis sheds light on evolutionary trajectories of young transcription start sites in the human genome
Source: Genome Res. 2018 May;28(5):676–88. doi: 10.1101/gr.231449.117 (PMC5932608; doi:10.1101/gr.231449.117)
Supplement: Supplemental Material [file supp_gr.231449.117_Supplemental_Fig_S5.pdf]

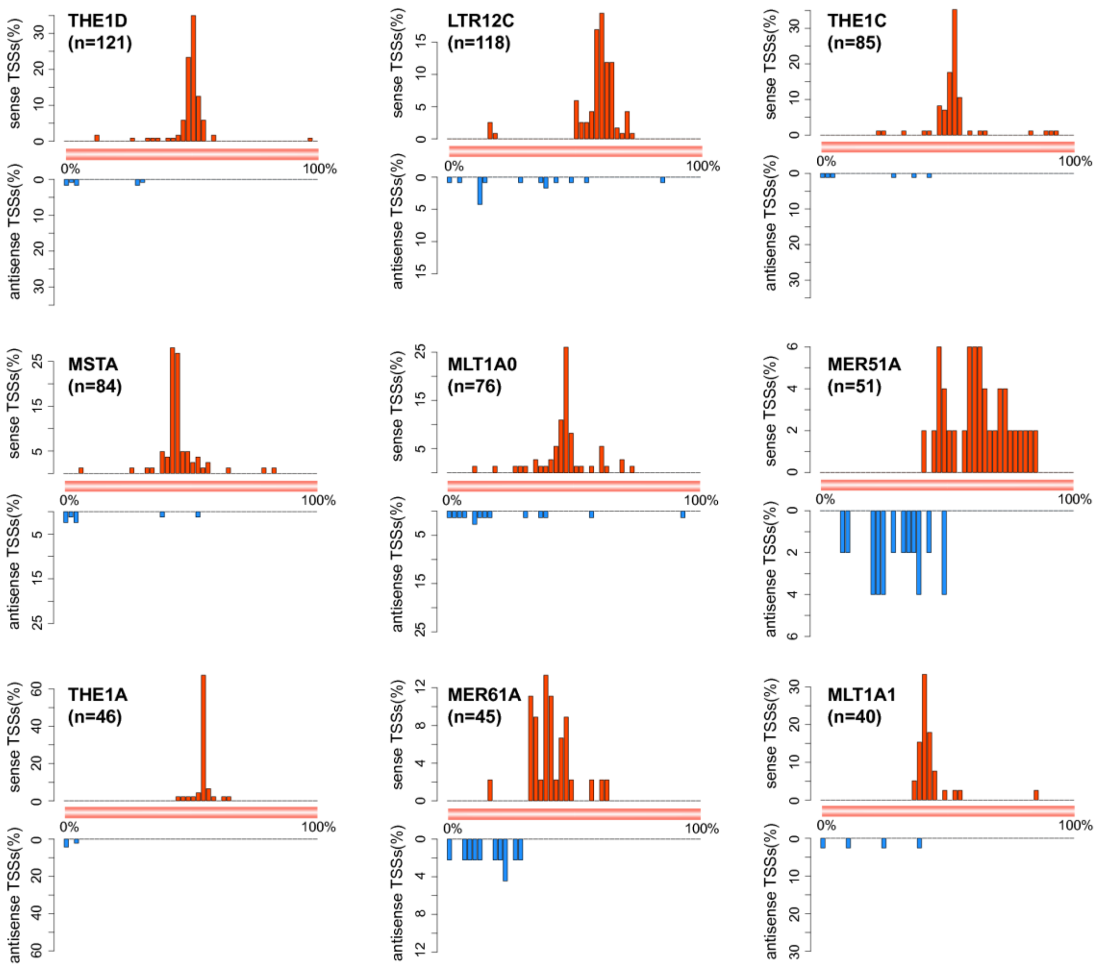

Supplemental Figure S5 Distribution of young TSSs along LTR subfamilies. These nine subfamilies are among the top 10 LTR subfamilies which harbor most young TSSs. The tenth, THEIB, has already been shown in **Fig. 2b**. Number of young TSSs for each subfamily is given in the bracket.
