# Supplementary material for: Integrated analysis sheds light on evolutionary trajectories of young transcription start sites in the human genome
Source: Genome Res. 2018 May;28(5):676–88. doi: 10.1101/gr.231449.117 (PMC5932608; doi:10.1101/gr.231449.117)
Supplement: Supplemental Material [file supp_gr.231449.117_Supplemental_Fig_S6.pdf]

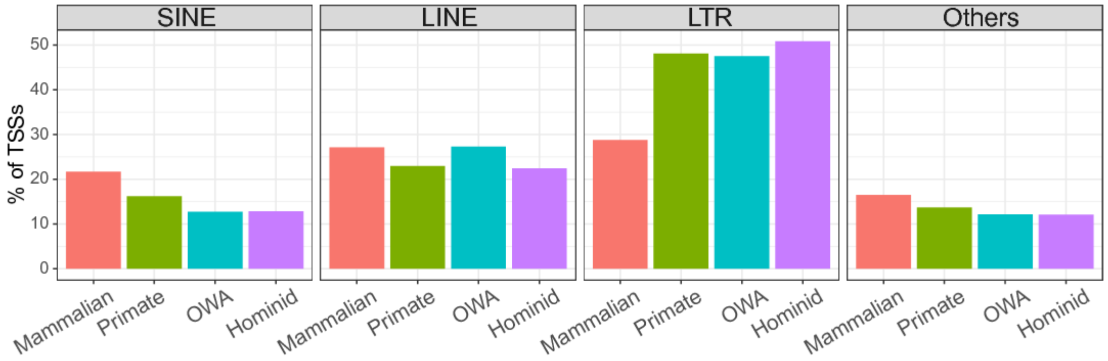

Supplemental Figure S6 Percentages of TSSs associated with different retrotransposons which contain a TATA-box motif starting at 25-35 bp upstream regions of the dominant TSSs.
