# Supplementary material for: Integrated analysis sheds light on evolutionary trajectories of young transcription start sites in the human genome
Source: Genome Res. 2018 May;28(5):676–88. doi: 10.1101/gr.231449.117 (PMC5932608; doi:10.1101/gr.231449.117)
Supplement: Supplemental Material [file supp_gr.231449.117_Supplemental_Fig_S7.pdf]

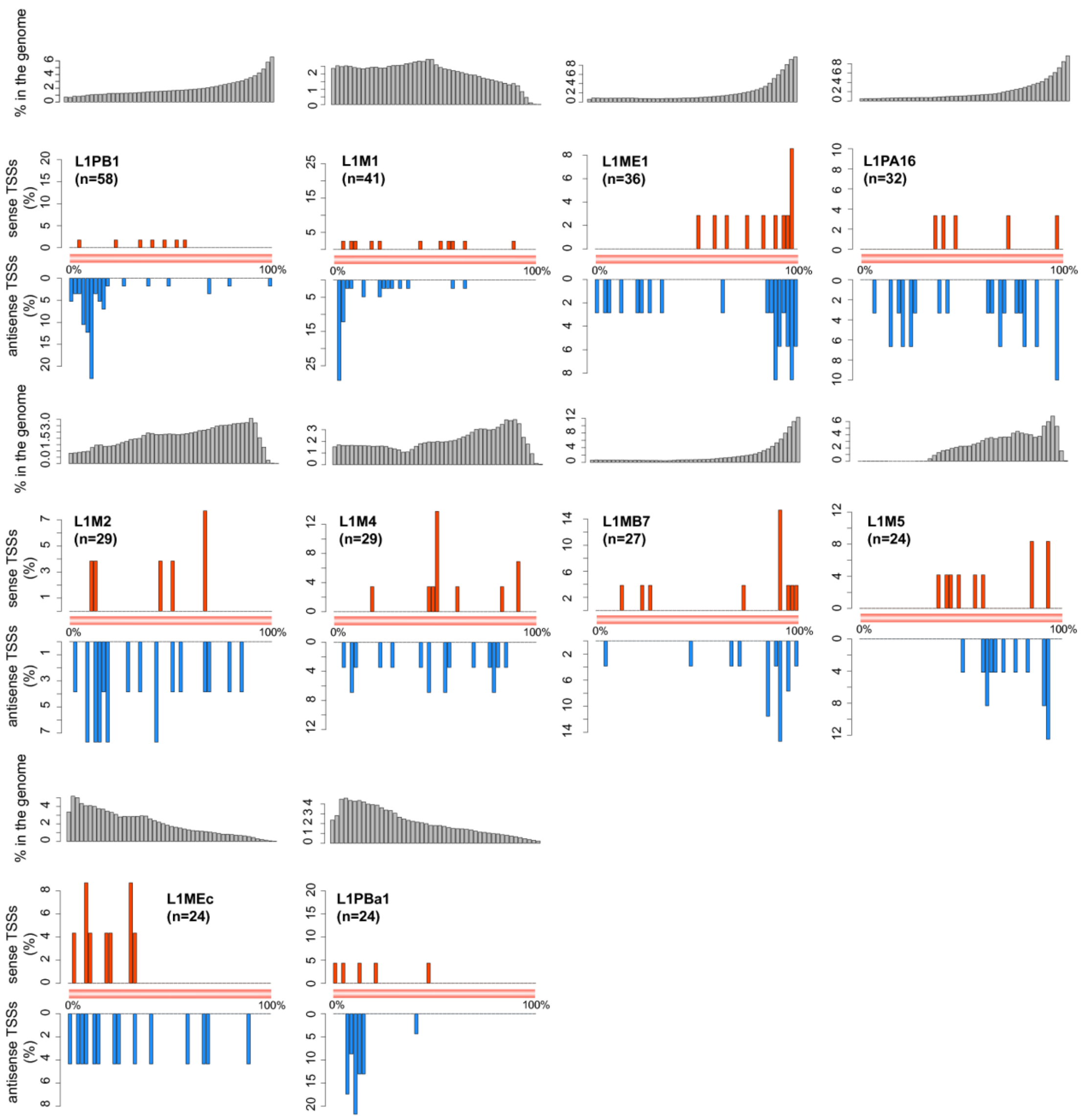

Supplemental Figure S7 Distribution of young TSSs along L1 subfamilies. The top 10 L1 subfamilies, which harbor most young TSSs, show considerable heterogeneity regarding the positions of young TSSs within the consensus sequences. The gray barplots are background positional distributions of sequences from the corresponding subfamilies in the human genome. Number of young TSSs for each subfamily is given in the bracket.
