# Supplementary material for: Integrated analysis sheds light on evolutionary trajectories of young transcription start sites in the human genome
Source: Genome Res. 2018 May;28(5):676–88. doi: 10.1101/gr.231449.117 (PMC5932608; doi:10.1101/gr.231449.117)
Supplement: Supplemental Material [file supp_gr.231449.117_Supplemental_Fig_S8.pdf]

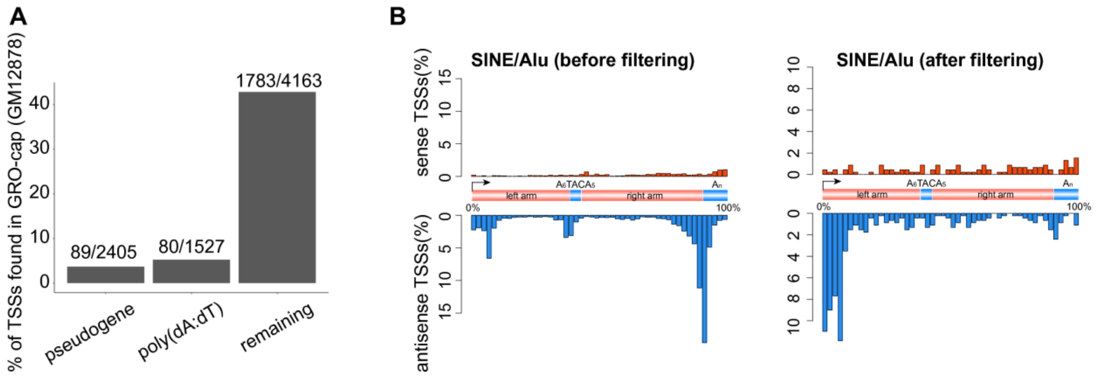

Supplemental Figure S8 Putative false positives associated with pseudogenes and poly(dA:dT) tracts in FANTOM 5 TSSs. (**A**) Percentages of FANTOM 5 TSSs of GM12878 found in GRO-cap defined TSSs of GM12878 (from Core et al. 2014), based on the FANTOM TSSs found only in primate lineages. A FANTOM TSS is considered to be found in the GRO-cap dataset if it is within 100 bp of a GRO-cap TSS. (**B**) Distribution of FANTOM 5 TSSs along the *Alu* consensus element before and after filtering the suspicious TSSs.
