# Supplementary material for: Integrated analysis sheds light on evolutionary trajectories of young transcription start sites in the human genome
Source: Genome Res. 2018 May;28(5):676–88. doi: 10.1101/gr.231449.117 (PMC5932608; doi:10.1101/gr.231449.117)
Supplement: Supplemental Material [file supp_gr.231449.117_Supplemental_Fig_S9.pdf]

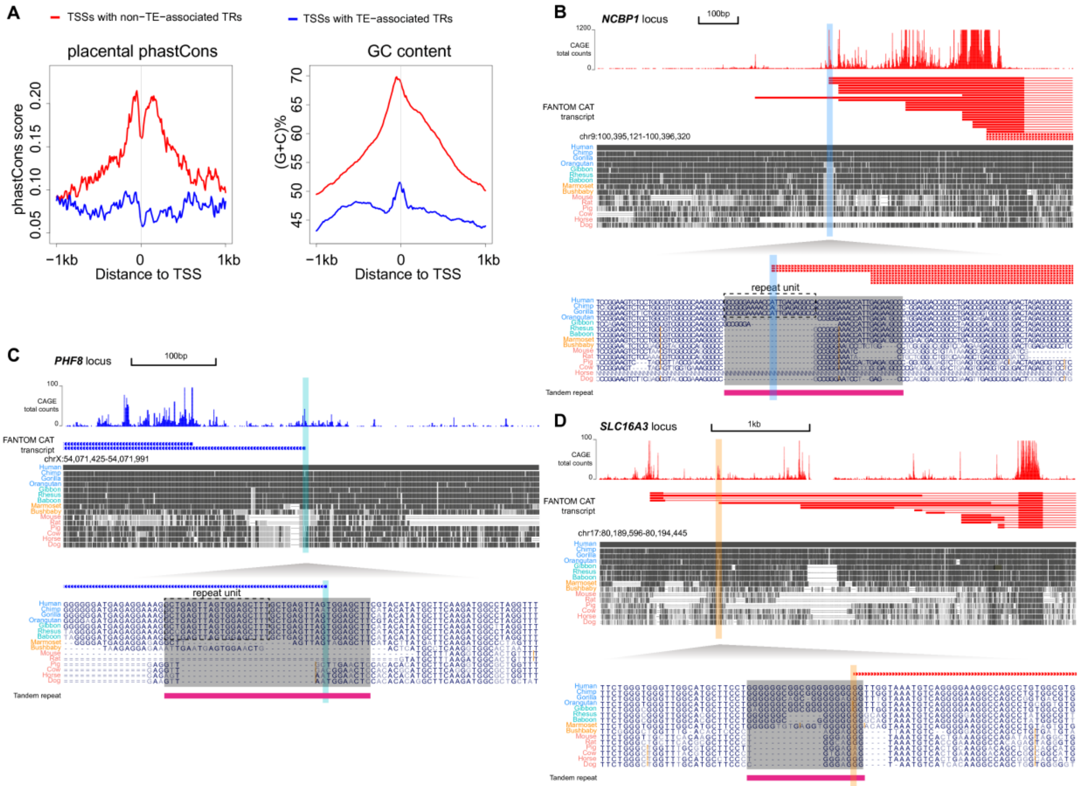

Supplemental Figure S9 TSSs associated with tandem repeats (TRs) but not associated with TEs. (**A**) Comparison of TSSs with non-TE-associated TRs and TSSs with TE-associated TRs regarding sequence conservation scores among placental mammals and GC content. (**B-D**) Examples of non-TE-associated TR expansions which contribute to new TSSs in (B) 'hominid', (C) 'OWA' and (D) 'primate' groups respectively. In each panel, at the top are the CAGE total tag counts and transcripts from FANTOM (red, forward strand; blue, reverse strand) and genomic alignment blocks from UCSC genome browser; at the bottom is the enlarged region of the young TSS, with grey shade indicating the TR expansion.
