# Supplementary material for: Integrated analysis sheds light on evolutionary trajectories of young transcription start sites in the human genome
Source: Genome Res. 2018 May;28(5):676–88. doi: 10.1101/gr.231449.117 (PMC5932608; doi:10.1101/gr.231449.117)
Supplement: Supplemental Material [file supp_gr.231449.117_Supplemental_Fig_S10.pdf]

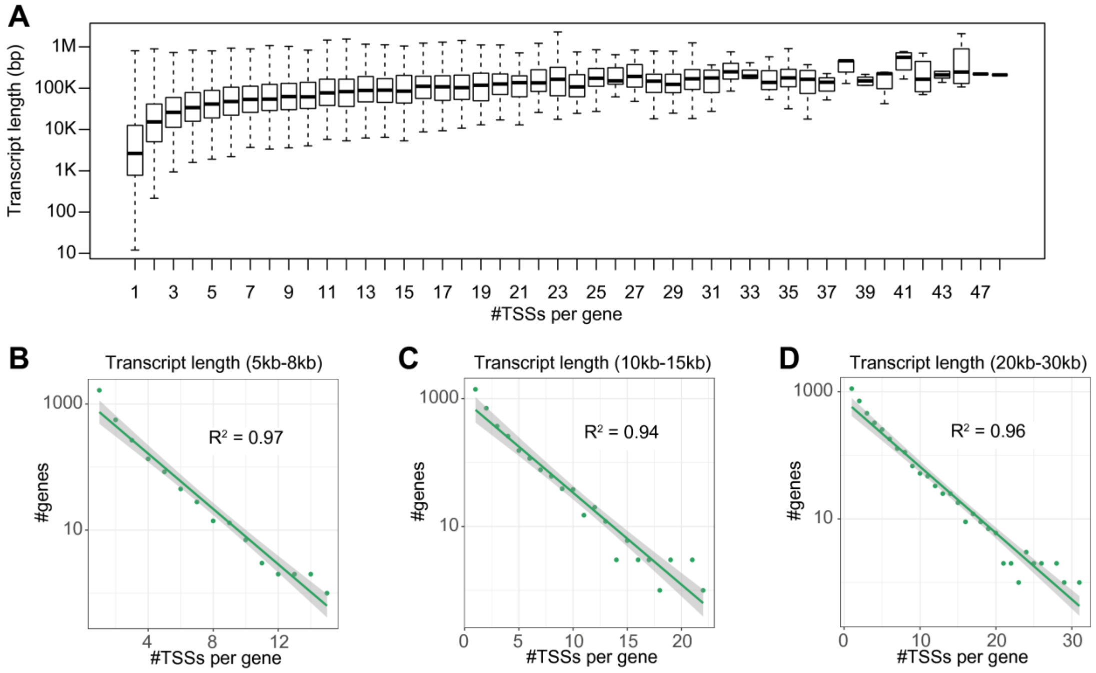

Supplemental Figure S10 The exponential relationship between number of genes with a specific number of TSSs and number of TSSs per gene is independent of the gene lengths. (**A**) Boxplots of transcript lengths for genes with different numbers of TSSs. For each gene, we used the length of its longest transcript. Although genes that have more TSSs tend to have longer transcripts, there are also many long genes that have small numbers of TSSs. Inspecting genes within specific length ranges (panels **B-D**), still reveals a clear exponential relationship. R^2 is the coefficient of determination for the linear regression in the figure.
