# Supplementary material for: Integrated analysis sheds light on evolutionary trajectories of young transcription start sites in the human genome
Source: Genome Res. 2018 May;28(5):676–88. doi: 10.1101/gr.231449.117 (PMC5932608; doi:10.1101/gr.231449.117)
Supplement: Supplemental Material [file supp_gr.231449.117_Supplemental_Fig_S11.pdf]

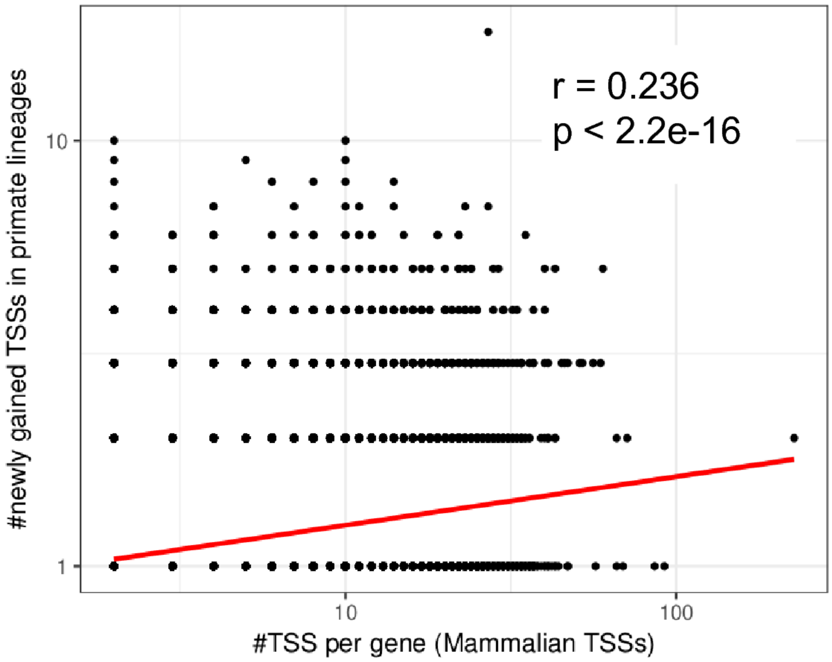

Supplemental Figure S11 Relationship between the number of old ('mammalian') TSSs per gene and the number of newly gained TSSs in primate lineages, on a log10 scale. The red line is derived from linear regression based on the data points. Pearson's r and the corresponding p-value are also shown in the figure.
