# Supplementary material for: Integrated analysis sheds light on evolutionary trajectories of young transcription start sites in the human genome
Source: Genome Res. 2018 May;28(5):676–88. doi: 10.1101/gr.231449.117 (PMC5932608; doi:10.1101/gr.231449.117)
Supplement: Supplemental Material [file supp_gr.231449.117_Supplemental_Fig_S12.pdf]

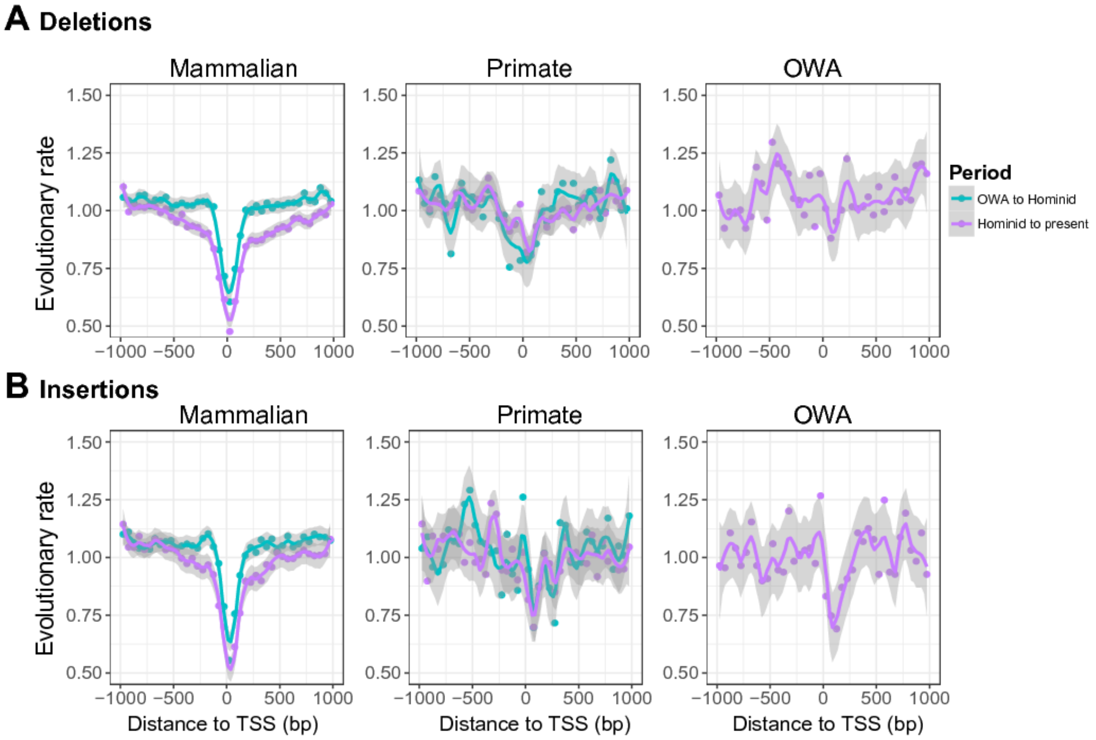

Supplemental Figure S12 Relative deletion and insertion rates (normalized by genomic average) inferred from genomic alignments for three TSS groups. Average rate were calculated for 40 bins along TSS±1kb. We estimated insertion/deletion rates of two periods for 'mammalian' and 'primate' groups, but only one for the 'OWA' group so as to focus on the evolutionary rates after TSS loci emerged in the genome. Fitting curves were estimated by 'loess' method.
