# Supplementary material for: Integrated analysis sheds light on evolutionary trajectories of young transcription start sites in the human genome
Source: Genome Res. 2018 May;28(5):676–88. doi: 10.1101/gr.231449.117 (PMC5932608; doi:10.1101/gr.231449.117)
Supplement: Supplemental Material [file supp_gr.231449.117_Supplemental_Fig_S13.pdf]

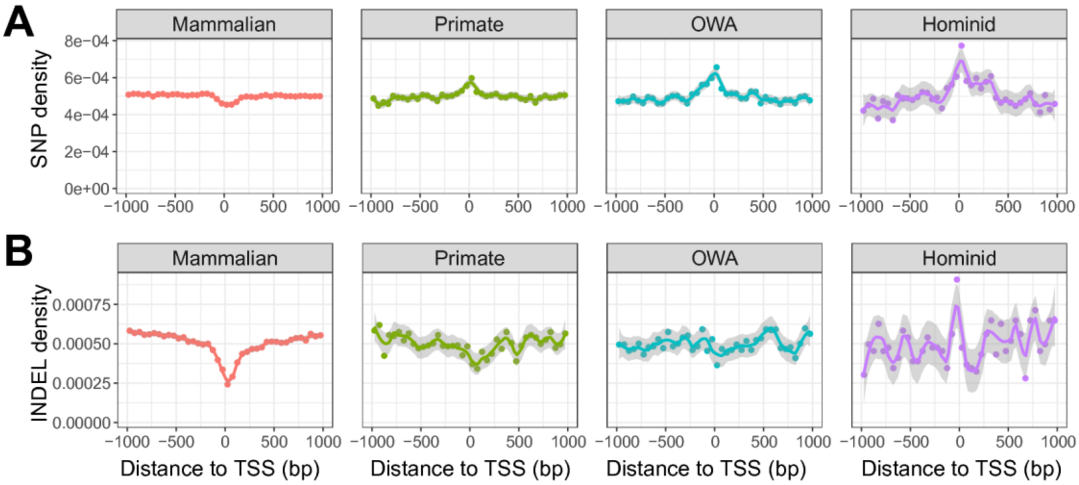

Supplemental Figure S13 Single nucleotide polymorphism (SNP, panel A) and insertion/deletion (INDEL, panel B) densities around TSSs (40 bins along TSS±1kb), based on variants of the 1000 genomes project phase 3 release. Only the biallelic variants with a minor allele frequency of ≥ 0.01 were considered. Because the genotype files in 1000 genomes project lack the ancestral allele information for insertion/deletion variants, the insertion and deletion variants were merged together for this analysis. Fitting curves were estimated by the ‘loess’ method.
