# Supplementary material for: Integrated analysis sheds light on evolutionary trajectories of young transcription start sites in the human genome
Source: Genome Res. 2018 May;28(5):676–88. doi: 10.1101/gr.231449.117 (PMC5932608; doi:10.1101/gr.231449.117)
Supplement: Supplemental Material [file supp_gr.231449.117_Supplemental_Fig_S14.pdf]

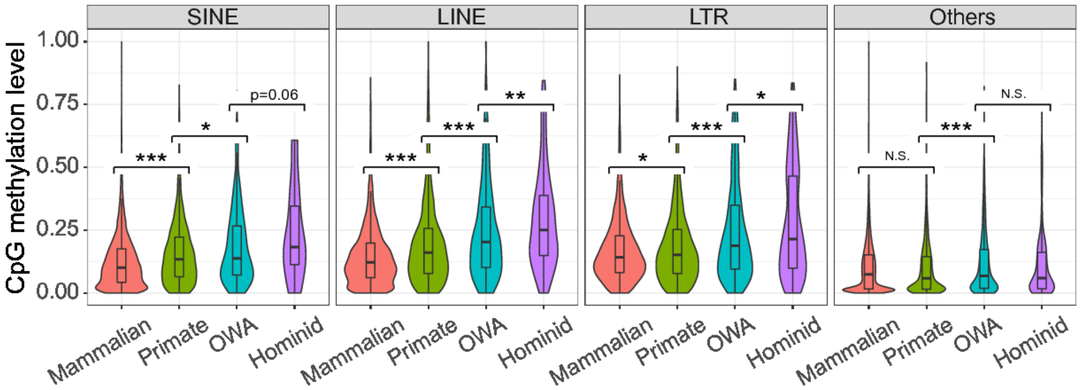

Supplemental Figure S14 DNA methylation in TSS loci in the germline. Violin and box plots for germline CpG methylation levels (data from Guo et al. 2015) in different TSS subgroups defined by the types of associated retrotransposons. For each TSS, average methylation level of CpGs in the 2 kb around the TSS was calculated. The TSSs in the "Others" group are mostly non-TE-associated TSSs, except for a few that are associated with DNA transposons. Statistical significance was calculated using the one tailed Wilcoxon rank sum test ("*", $p < 0.05$; "**", $p < 0.01$; "***", $p < 0.001$; N.S., not significant).
