# Supplementary material for: Integrated analysis sheds light on evolutionary trajectories of young transcription start sites in the human genome
Source: Genome Res. 2018 May;28(5):676–88. doi: 10.1101/gr.231449.117 (PMC5932608; doi:10.1101/gr.231449.117)
Supplement: Supplemental Material [file supp_gr.231449.117_Supplemental_Fig_S15.pdf]

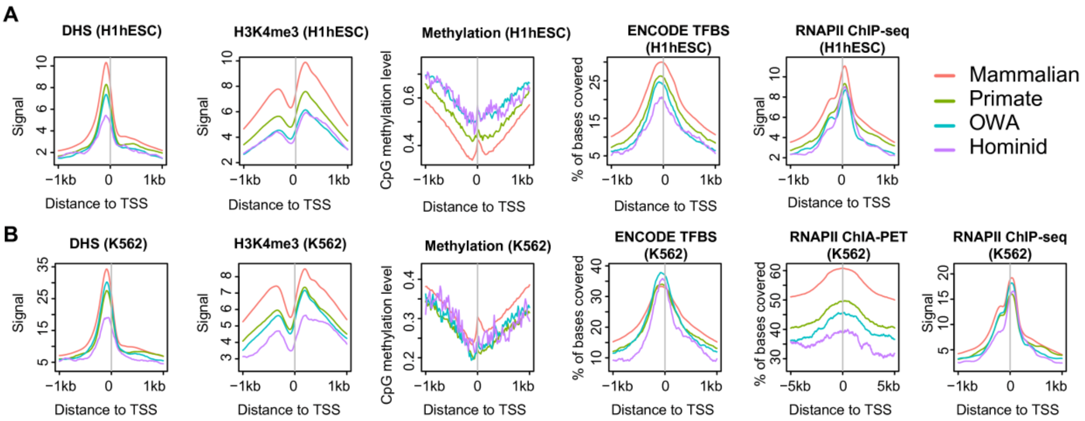

Supplemental Figure S15 (**A**) Meta-profiles of functional signatures in H1-hESC cell line in different TSS groups. (**B**) Meta-profiles of functional signatures in K562 cell line in different TSS groups. Global hypomethylation in the K562 cell line has been previously reported, so the similar pattern of DNA methylation meta-profiles in K562 across TSS groups is not surprising. For the TFBS analysis, we merged the called peaks of TF ChIP-seq datasets and calculated how many bases around TSSs are covered by the peaks. The figure for RNAP II ChIA-PET in H1-hESC is missing because of lack of publicly available data.
