# Supplementary material for: Integrated analysis sheds light on evolutionary trajectories of young transcription start sites in the human genome
Source: Genome Res. 2018 May;28(5):676–88. doi: 10.1101/gr.231449.117 (PMC5932608; doi:10.1101/gr.231449.117)
Supplement: Supplemental Material [file supp_gr.231449.117_Supplemental_Fig_S16.pdf]

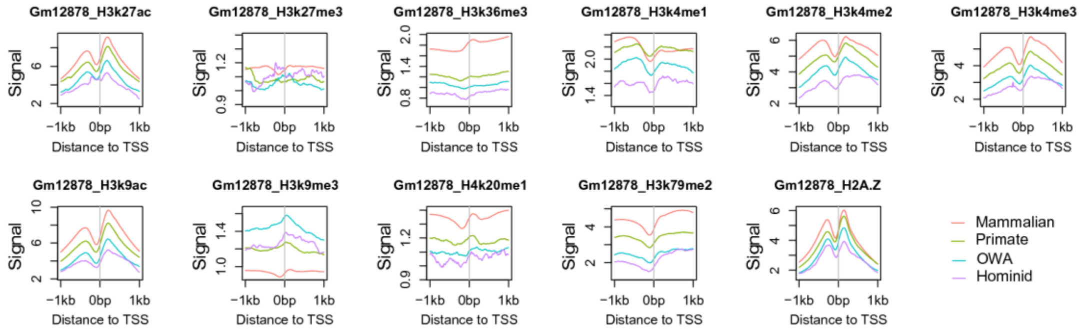

Supplemental Figure S16 Meta-profiles for histone modifications in GM12878, supplementary to that shown in Fig. 4. All the data was obtained from ENCODE project.
