# Supplementary material for: Integrated analysis sheds light on evolutionary trajectories of young transcription start sites in the human genome
Source: Genome Res. 2018 May;28(5):676–88. doi: 10.1101/gr.231449.117 (PMC5932608; doi:10.1101/gr.231449.117)
Supplement: Supplemental Material [file supp_gr.231449.117_Supplemental_Fig_S17.pdf]

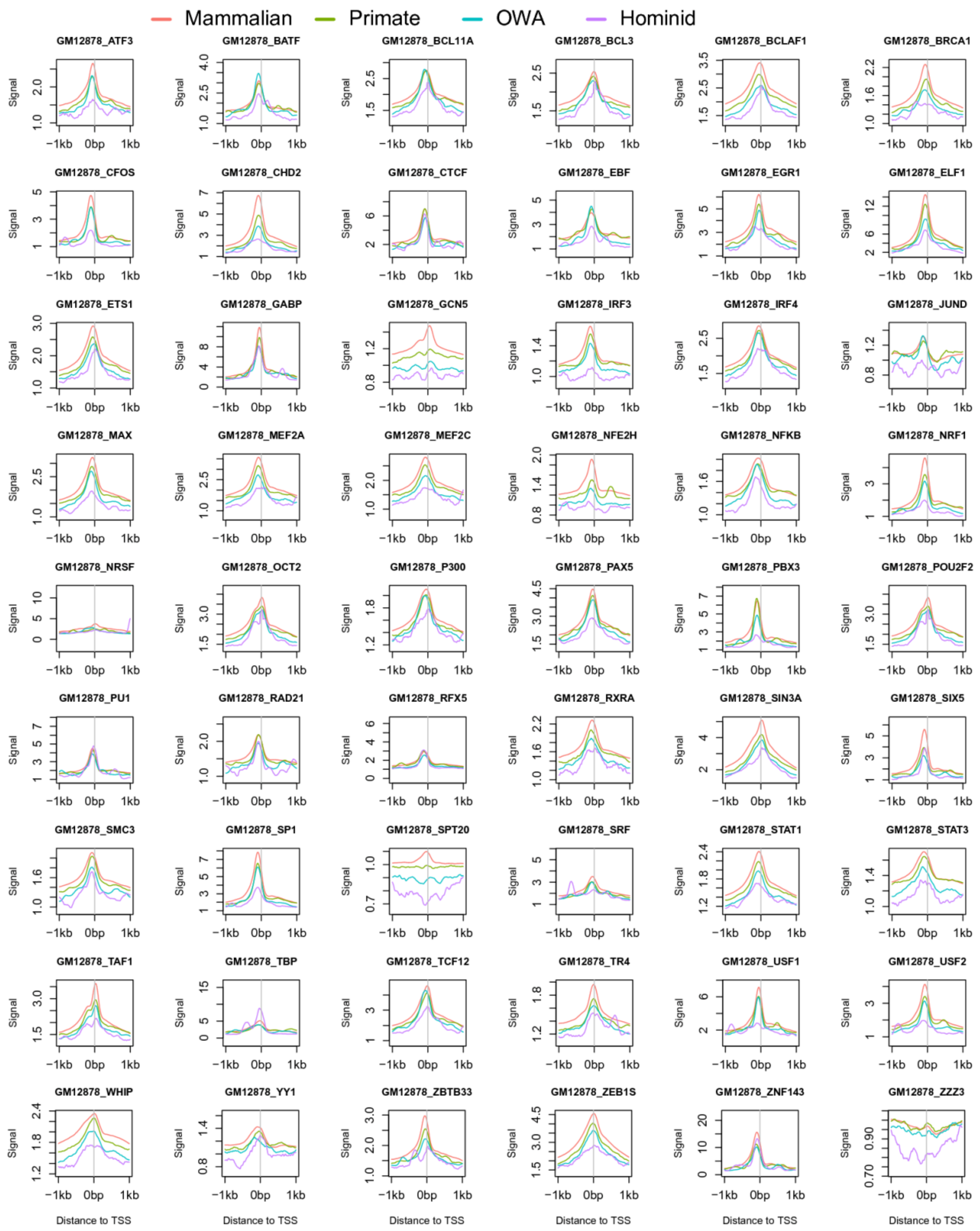

Supplemental Figure S17 Meta-profiles for TF ChIP-seq signals in GM12878 cell line in different TSS groups. All the data was obtained from ENCODE project.
