# Supplementary material for: Integrated analysis sheds light on evolutionary trajectories of young transcription start sites in the human genome
Source: Genome Res. 2018 May;28(5):676–88. doi: 10.1101/gr.231449.117 (PMC5932608; doi:10.1101/gr.231449.117)
Supplement: Supplemental Material [file supp_gr.231449.117_Supplemental_Fig_S18.pdf]

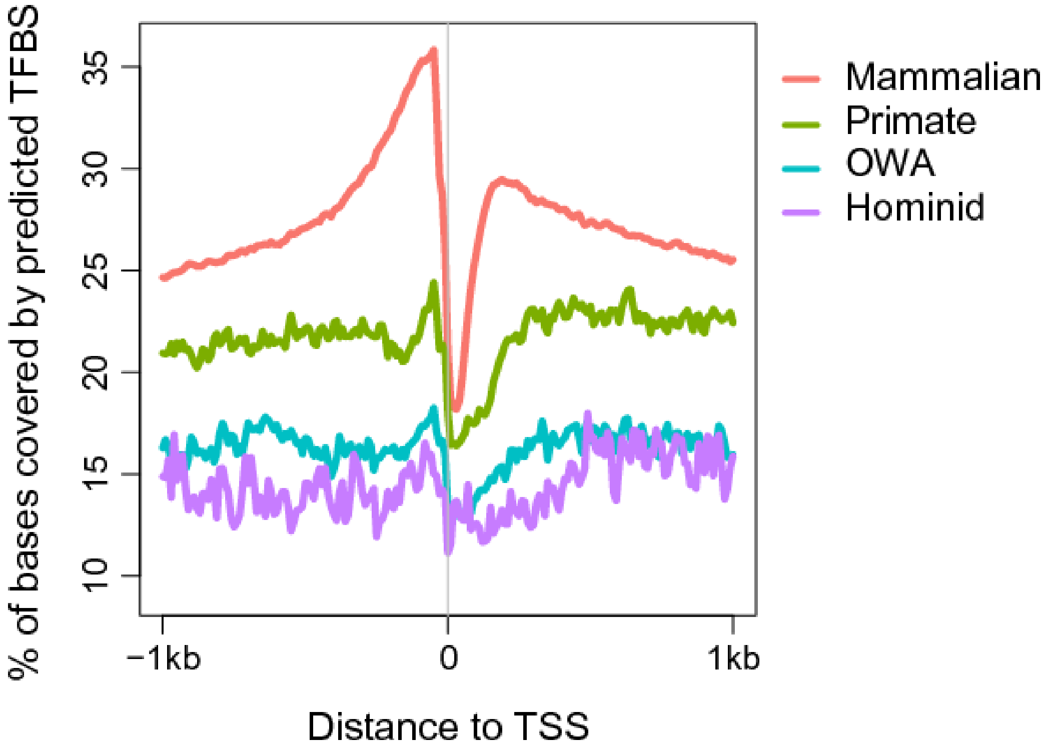

Supplemental Figure S18 Comparison of the coverage by computationally predicted TFBSs between four TSS groups. The computationally predicted TFBSs in human genome were from ENCODE project (http://compbio.mit.edu/encode-motifs/). Note that the TFBSs predicted by computational methods are based on binding motifs, usually smaller than the called peaks in the TF ChIP-seq data that were used for generating **Fig. 4**.
