# Supplementary material for: Integrated analysis sheds light on evolutionary trajectories of young transcription start sites in the human genome
Source: Genome Res. 2018 May;28(5):676–88. doi: 10.1101/gr.231449.117 (PMC5932608; doi:10.1101/gr.231449.117)
Supplement: Supplemental Material [file supp_gr.231449.117_Supplemental_Fig_S19.pdf]

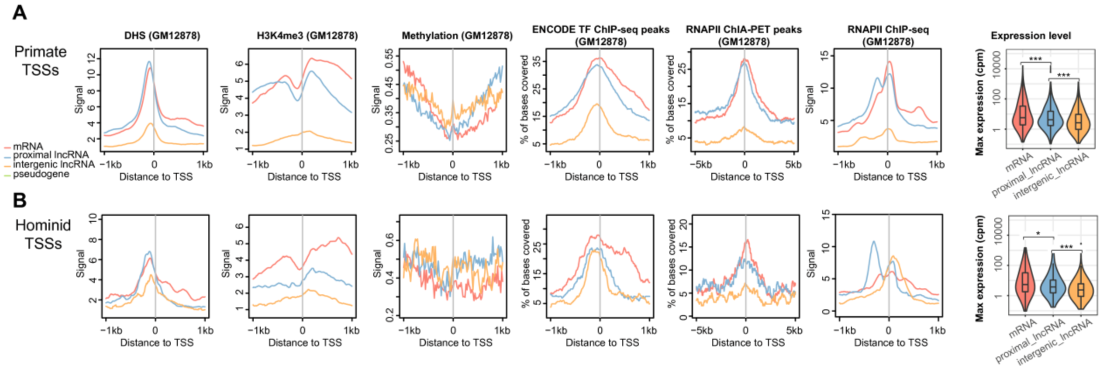

Supplemental Figure S19 Meta-profiles of functional signatures in GM12878 cell line for different TSS subgroups, defined by transcript types. (**A**) For 'primate' TSS subgroups. (**B**) For 'hominid' TSS subgroups. Statistical significance was calculated using the one-tailed Wilcoxon rank sum tests ("*", $p < 0.05$; "**", $p < 0.01$; "***", $p < 0.001$).
