# Supplementary material for: Integrated analysis sheds light on evolutionary trajectories of young transcription start sites in the human genome
Source: Genome Res. 2018 May;28(5):676–88. doi: 10.1101/gr.231449.117 (PMC5932608; doi:10.1101/gr.231449.117)
Supplement: Supplemental Material [file supp_gr.231449.117_Supplemental_Fig_S20.pdf]

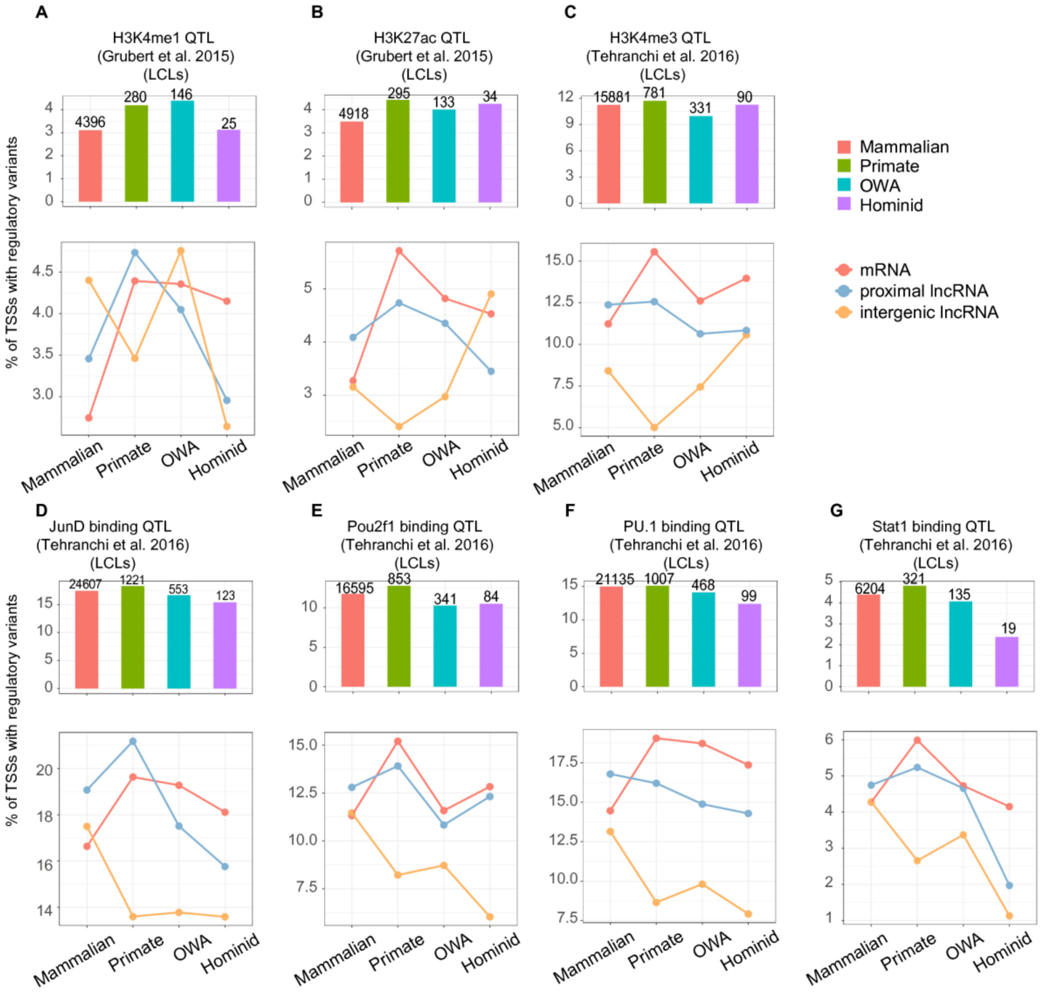

Supplemental Figure S20 Proportions of TSSs harboring regulatory variants within TSS±1kb in different TSS groups in additional datasets. The results in this figure were based on regulatory variants with derived allele frequency (DAF) ≥ 0.01. Above the bars are the numbers of TSSs with regulatory variants. Note that for the QTL dataset from Grubert et al. (2015), the numbers of regulatory variants found in the TSS groups/subgroups are quite small, so the trends shown in the panels **A-B** for different transcript types may not accurately reflect actual trends. LCLs, lymphoblastoid cell lines.
