# Supplementary material for: Integrated analysis sheds light on evolutionary trajectories of young transcription start sites in the human genome
Source: Genome Res. 2018 May;28(5):676–88. doi: 10.1101/gr.231449.117 (PMC5932608; doi:10.1101/gr.231449.117)
Supplement: Supplemental Material [file supp_gr.231449.117_Supplemental_Fig_S21.pdf]

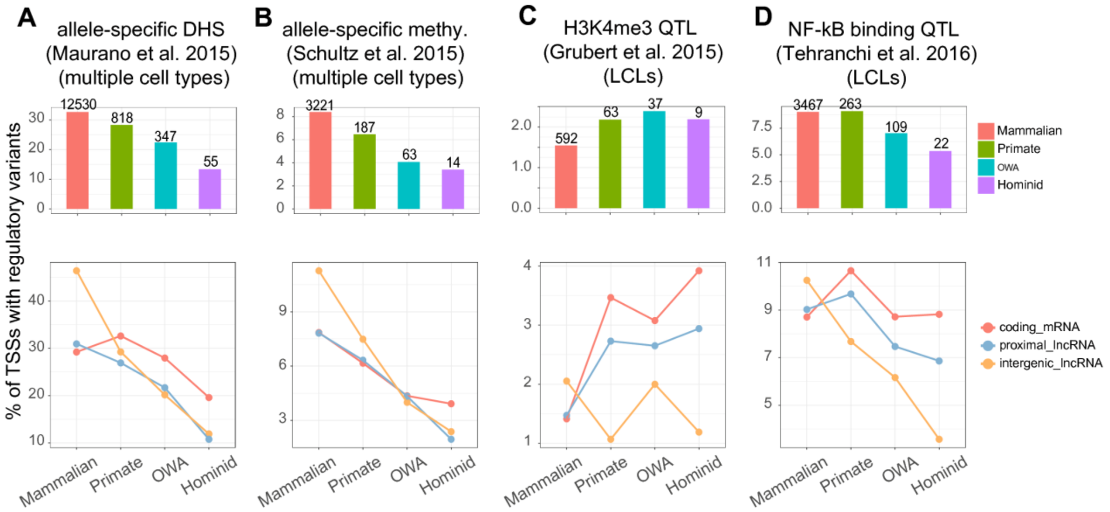

Supplemental Figure S21 Proportions of TSSs harboring regulatory variants within TSS±1kb in different TSS groups excluding the TSSs separated by < 2 kb. The shown results are based on variants with DAF ≥ 0.01. Above the bars are the numbers of TSSs with regulatory variants. Note that for the H3K4me3 QTL dataset from Grubert et al. (2015), the numbers of regulatory variants found in the TSS groups/subgroups are very small, so the changing trends shown in the panel **C** for different transcript types may not accurately reflect actual trends. LCLs, lymphoblastoid cell lines.
