# Supplementary material for: Integrated analysis sheds light on evolutionary trajectories of young transcription start sites in the human genome
Source: Genome Res. 2018 May;28(5):676–88. doi: 10.1101/gr.231449.117 (PMC5932608; doi:10.1101/gr.231449.117)
Supplement: Supplemental Material [file supp_gr.231449.117_Supplemental_Fig_S22.pdf]

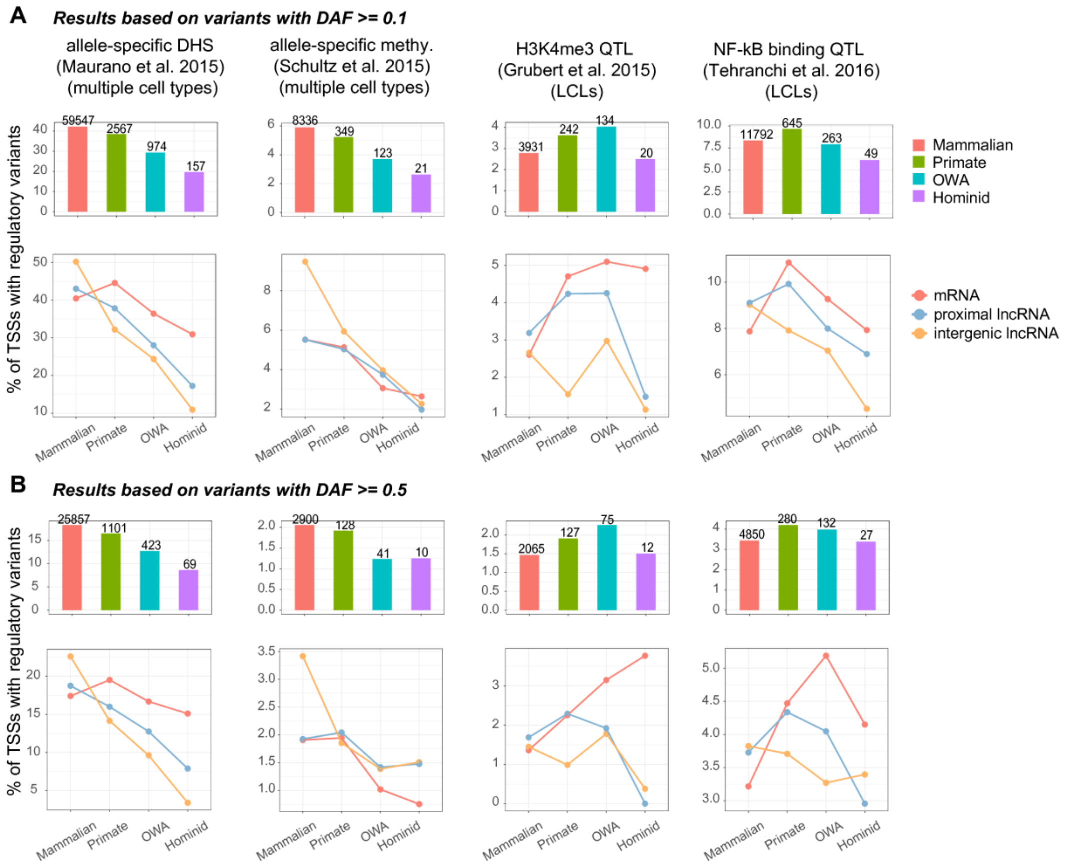

Supplemental Figure S22 Proportions of TSSs harboring regulatory variants within TSS±1kb in different TSS groups, based on variants with higher thresholds of derived allele frequency (DAF). (**A**) Results based on variants with DAF ≥ 0.1. (**B**) Results based on variants with DAF ≥ 0.5. Above the bars are the numbers of TSSs with regulatory variants. Note that for the results based on variants with DAF ≥ 0.5, the numbers of regulatory variants found in the TSS groups/subgroups are very small, so the changing trends shown in the some panels for different transcript types may not accurately reflect actual trends. LCLs, lymphoblastoid cell lines.
