# Supplementary material for: Integrated analysis sheds light on evolutionary trajectories of young transcription start sites in the human genome
Source: Genome Res. 2018 May;28(5):676–88. doi: 10.1101/gr.231449.117 (PMC5932608; doi:10.1101/gr.231449.117)
Supplement: Supplemental Material [file supp_gr.231449.117_Supplemental_Fig_S23.pdf]

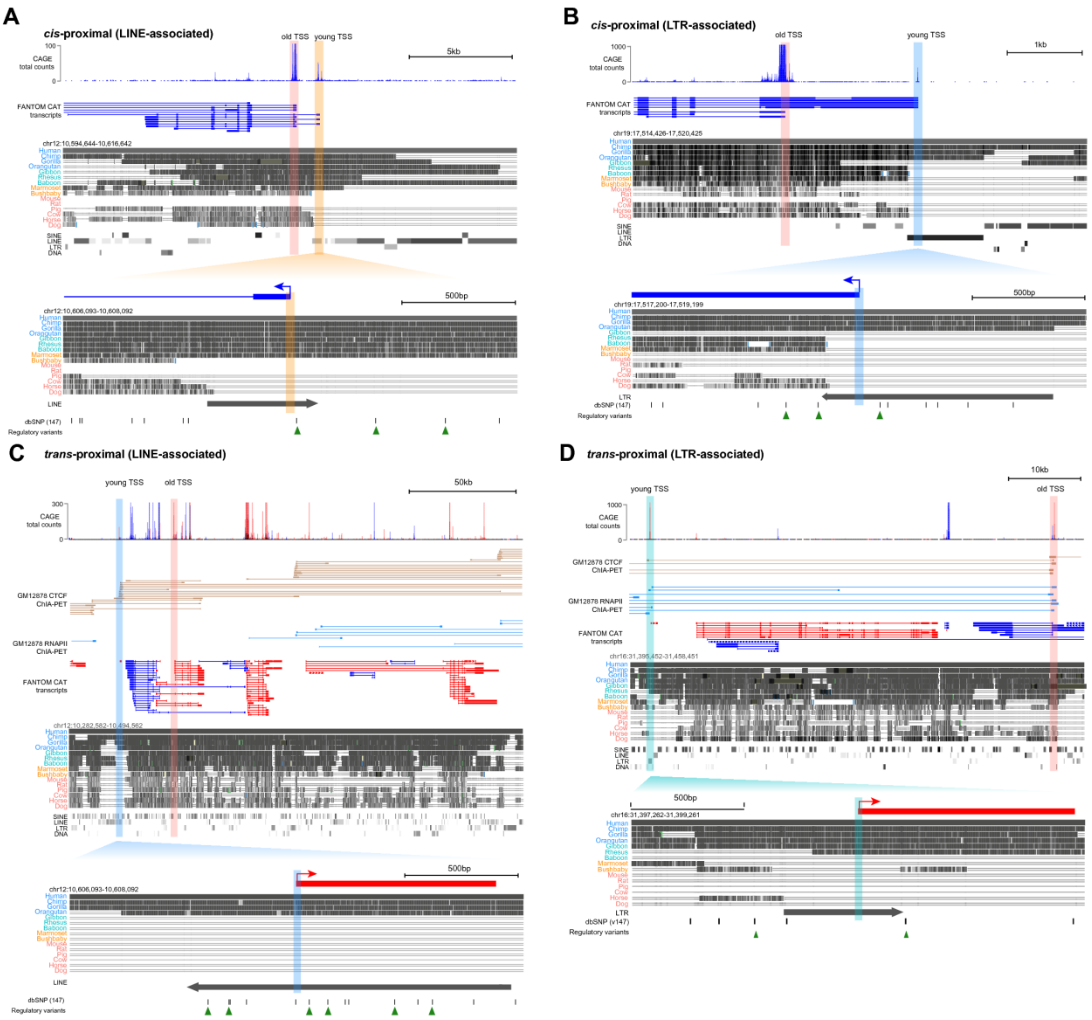

Supplemental Figure S23 Additional examples for *cis*-proximal and *trans*-proximal young TSSs. In each panel, from top to bottom: 1) CAGE total tag counts from FANTOM; 2) CTCF and RNAP II ChIA-PET interactions (only for *trans*-proximal examples); 3) FANTOM CAT transcript models, red for forward-strand and blue for reverse-strand transcripts; 4) genome alignments represented by grey blocks and transposable elements within this region, generated from UCSC genome browser; 5) the enlarged region of the young TSS. The old and young TSSs are indicated with shades of different colors (red, “mammalian”; green, “primate”; cyan, “OWA”; blue, “hominid”). The positions of regulatory variants are shown with small triangles in the enlarged figures.
